# Associations between compliance with covid-19 public health recommendations and perceived contagion in others: a self-report study in Swedish university students

**DOI:** 10.1186/s13104-021-05848-6

**Published:** 2021-11-25

**Authors:** Claes Andersson, Marcus Bendtsen, Olof Molander, Lilian Granlund, Naira Topooco, Karin Engström, Petra Lindfors, Anne H. Berman

**Affiliations:** 1 Department of Criminology, Malmö University, Malmö, Sweden; 2 Department of Psychology, Uppsala University, Uppsala, Sweden; 3 Department of Health, Medicine and Caring Sciences, Linköping University, Linköping, Sweden; 4 Centre for Psychiatry Research, Department of Clinical Neuroscience, Karolinska Institutet, & Stockholm Health Care Services, Region Stockholm, Stockholm, Sweden; 5 Department of Behavioral Sciences and Learning, Linköping University, Linköping, Sweden; 6 Department of Global Public Health, Karolinska Institutet, Stockholm, Sweden; 7 Department of Psychology, Stockholm University, Stockholm, Sweden

**Keywords:** COVID-19, University students, Contagion in others, Public health recommendations, Bayesian inference

## Abstract

**Objective:**

During the COVID pandemic, government authorities worldwide have tried to limit the spread of the virus. Sweden’s distinctive feature was the use of voluntary public health recommendations. Few studies have evaluated the effectiveness of this strategy. Based on data collected in the spring of 2020, this study explored associations between compliance with recommendations and observed symptoms of contagion in others, using self-report data from university students.

**Results:**

Compliance with recommendations ranged between 69.7 and 95.7 percent. Observations of moderate symptoms of contagion in “Someone else I have had contact with” and “Another person” were markedly associated with reported self-quarantine, which is the most restrictive recommendation, complied with by 81.2% of participants. Uncertainty regarding the incidence and severity of contagion in cohabitants was markedly associated with the recommendation to avoid public transportation, a recommendation being followed by 69.7%. It is concluded that students largely followed the voluntary recommendations implemented in Sweden, suggesting that coercive measures were not necessary. Compliance with recommendations were associated with the symptoms students saw in others, and with the perceived risk of contagion in the student’s immediate vicinity. It is recommended that voluntary recommendations should stress personal relevance, and that close relatives are at risk.

**Supplementary Information:**

The online version contains supplementary material available at 10.1186/s13104-021-05848-6.

## Introduction

When the COVID-19-pandemic spread globally in early 2020, governments urgently sought to introduce measures to limit the spread and contagion of the virus by reducing social contacts. Due to the constitution, Swedish authorities did not have the option of closing activities nor of forcibly isolating people. Instead, a policy of public health recommendations based on trust was introduced [6]. This approach is based on Self-Determination Theory (SDT), where authorities set limits in an autonomy-supportive, caring, and competence-fostering way to achieve adherence and long-term persistence [8]. Sweden’s choice of route makes it possible to study whether voluntary strategies might be as effective as the mandatory strategies that were simultaneously introduced in several other countries. Considering both current and possible future pandemics, knowledge about factors having an impact on compliance with voluntary public health recommendations is important so that recommendations can be designed efficiently.

It has been suggested that public health recommendations are more likely to be followed if connected to important values and aspirations of the individual [8]. Compliance is also known to be associated with individuals’ observed perceptions of risk, i.e., assessment of the probability and severity of harm in relation to the possible gains offered by following recommendations [14]. Risks are perceived as more threatening the closer the relationship is to a potential threat [9]. Uncertainty regarding the severity of the threat has been associated with lower engagement in risk-reducing behavior [11]. Risk perception also tends to be relatively low in young adults [13]. Recent research during the COVID-19 pandemic shows that risk perception correlates with adoption of preventive health behaviors [5]. In young adults, self-perceived risk, together with the desire to protect others, has been associated with adherence to preventive measures [15]. In OECD countries, approximately half of the young adult population consists of university students [10]. To our knowledge, there are no reports on the associations between compliance with public health recommendations and perceived contagion among others, among university students.

## The present study

With the overall objective to study recommendation compliance, contagion, and mental health effects of the COVID-19 pandemic among university students in Sweden, a longitudinal questionnaire study was initiated. The study included a baseline assessment in May 2020, and follow-up assessments in the fall of 2020, and in the spring of 2021. The survey was developed for the current study (see Additional file 16).

The present study uses secondary self-report data from the baseline assessment that were part of the original analysis plan [1]. Previously associations between compliance with public health recommendations and self-reported symptoms, mental health, and academic self-efficacy have been reported [2]. The current study aims to identify whether compliance with public health recommendations is associated with observed symptoms of contagion in others. It is hypothesized that recommendation compliance will be associated with student’s observations of symptoms within society, as well as with risk of contagion in the student's immediate vicinity.

## Main text

### Methods

The survey was conducted in May–June 2020, i.e., during the first wave of the COVID-19 pandemic. Participants were recruited through advertisements on websites of 10 Swedish universities and that of the national association of student unions in Sweden. Those who responded to the advertisement used URL or QR-code to access detailed project information for informed consent, after which a short web-survey was accessed. A total of 4495 students from 19 universities consented. Of the respondents, 70.9% were women, with a mean age of 26.5 (SD 5.27).

Compliance with the following public health recommendations was assessed: handwashing with soap/alcohol; remaining at home or self-quarantine, sneezing/coughing in one’s arm; keeping a distance from others when going out; avoiding meeting with persons who are older/in a risk group; avoiding traveling with public transportation; avoiding travel to other places in the country. Next, respondents were asked to report contagion, including severity of contagion, in the following categories: a person I live with, a person in my family (not living with); a person in my circle of acquaintances; someone I have had contact with; another person. For each category responses were given on the following scale: no symptoms; mild symptoms; moderate symptoms; severe symptoms; died; not relevant/do not know. For all questions, the respondent was asked to report on experiences from the last 4 weeks.

The study aim was addressed by estimating associations among variables using multinomial regression. The compatibility of coefficients’ values with respect to the data was estimated using Bayesian inference with both standard normal priors and regularizing priors. As several covariates were included in the model, the use of regularizing priors ensured a skeptical approach, pulling coefficients towards the null unless the data strongly suggested otherwise [4]. We complemented our Bayesian analyses with maximum likelihood estimates and null-hypothesis testing. All models included age and gender as covariates.

### Results

Compliance with the public health recommendations was highest for handwashing (95.7%) and lowest for avoiding public transportation (69.7%), see Table 1. Table 2 presents observed symptoms of contagion in others known to the respondent, as reported by the respondents. In assessed categories, absence of symptoms ranged between 39.9 and 59.9%. Mild to moderate symptoms were most frequent in the respondent’s circle of acquaintances, and deaths were most frequent in the category of others known to the respondent (5.7%). The proportions of “Not relevant/do not know” responses ranged between 11.9 and 44.7%.

**Table 1:** Compliance

Compliance	Compliant	Non-compliant
Washed hands	3931 (95.7%)	175 (4.3%)
Stayed home	3333 (81.2%)	770 (18.8%)
Sneezed/coughed in arm	3835 (93.7%)	258 (6.3%)
Kept distance	3578 (87.2%)	526 (12.8%)
Avoided risk groups	3917 (95.5%)	184 (4.5%)
Transportation	2856 (69.7%)	1240 (30.3%)
Travel	3538 (86.6%)	549 (13.4%)

**Table 2:** Symptoms of contagion in others

Symptoms of others	No symptoms	Mild symptoms	Moderate symptoms	Severe symptoms	Died	Not relevant/do not know
Living with	2090 (59%)	470 (13.3%)	179 (5.1%)	20 (0.6%)	0 (0%)	782 (22.1%)
Family	2133 (59.9%)	450 (12.6%)	340 (9.6%)	83 (2.3%)	22 (0.6%)	532 (14.9%)
Acquaintance	1266 (35.1%)	725 (20.1%)	892 (24.7%)	236 (6.5%)	60 (1.7%)	430 (11.9%)
Contact with	1580 (44.8%)	375 (10.6%)	326 (9.2%)	166 (4.7%)	73 (2.1%)	1008 (28.6%)
Other	1033 (39.9%)	57 (2.2%)	109 (4.2%)	86 (3.3%)	148 (5.7%)	1156 (44.7%)

Additional file 11: Fig. S1, Additional file 12: Fig. S2, Additional file 13: Fig. S3, Additional file 14: Fig. S4, Additional file 15: Fig. S5 show the marginal posterior distributions of coefficients in the multinomial regression models, representing associations between recommendation compliance and symptoms of contamination in others. Additional file 1: Table S1, Additional file 2: Table S2, Additional file 3: Table S3, Additional file 4: Table S4, Additional file 5: Table S5, Additional file 6: Table S6, Additional file 7: Table S7, Additional file 8: Table S8, Additional file 9: Table S9, Additional file 10: Table S10 provide numerical details. The below results present associations identified when applying regularizing priors.

Compliance with the public health recommendation to remain at home or self-quarantine, i.e., the most restrictive public health recommendation, was markedly associated with reports of moderate symptoms of contagion in the categories “Someone else I have had contact with” and “Another person”. Avoiding public transportation, i.e., the recommendation followed to the least extent, was markedly associated with responding “Not relevant/do not know” in relation to contamination in cohabitants.

### Discussion

When interpreting the results of Bayesian inference with regularizing priors, this study found support for the initial hypothesis, i.e., that recommendation compliance would be associated with students’ observations of symptoms in general society, as well as risk of contagion in the student’s immediate vicinity. Clearly, observations of symptoms in “Someone else I have had contact with” and “Another person” were associated with self-quarantine among university students in Sweden. This suggests that personal relevance may influence students to adopt such strict measures as going into self-quarantine to limit the spread and contagion of the COVID-19 virus. Voluntary self-quarantine is here connected to values and aspirations important to students [8].

Uncertainty regarding contagion in cohabitants was associated with compliance with the recommendation to avoid public transportation, meaning that respondents who presumably felt uncertain about whether they might be contagious, avoided public transportation to a larger extent than usual. Here too, personal relevance and concern for others seems to have positively influenced recommendation-compliant behavior. It should be noted that further associations in support of the hypothesis were identified but did not stand the test of using strict Bayesian regularizing priors (see Additional file 16).

In relation to symptoms in others, the high proportions of “Not relevant/do not know” responses probably reflect the prevailing uncertainty concerning contagion and the spread of the virus during the spring of 2020, not the least due to the lack of effective tests. Regarding the relatively low compliance in relation to avoiding public transport, the interpretation is that circumstances in Sweden play role: public transportation is affordable, well organized and easily accessible and thus many rely on it for daily commute. For many university students it is possibly the only available means of transport. To influence the propensity to use public transport, uncertainty regarding contamination in the respondent’s immediate vicinity seems to be required. Clearly recommendations seem to be followed when identified as important to protect those who are valuable [8].

In contrast to research suggesting that risk perceptions tend to be low in young adults [13], the present study found that compliance with public health recommendations was good, ranging between 69.7 and 95.7% during the initial phase of the pandemic. The overall high range of compliance suggests that the implementation of public-health recommendations to limit the spread and contagion of the COVID-19 virus was reasonably accepted among university students. These results are in line with research from countries that have applied stricter restrictions, and that also show that compliance with public health recommendations has been high during the ongoing COVID-19 pandemic [5, 15].

The Swedish way of dealing with the COVID-19 pandemic has gained international attention. The Swedish approach is based on the constitution, which draws on liberal ideas of citizens' freedoms and rights during peace, and make it difficult to impose legal restrictions. However, our findings suggest that, in the beginning of a pandemic, people are willing to accept public health recommendations if these are perceived as personally relevant. The results reported in this study can thus be used as an impetus to improve compliance with recommendations. In practice, this means that communication regarding public health recommendations might be developed to achieve further effect. For example, a practical implication, drawing on our findings, is that public health recommendations should aim to provide clear evidence of contagion in society, and point out the risk of contagion in close relatives to influence desired behavior/voluntary compliance.

For future research, behavioral economics offers a perspective that integrates economics and psychology for understanding behavior as well as how to influence it and would be helpful in extending the impact of existing communication strategies regarding recommendation compliance. Behavioral economics publications have been increasingly focused in recent years on strategies for changing unhealthy behaviors, on the national, community, and individual levels. One example of a behavioral economics strategy is to use “episodic future thinking” across multiple behaviors to vividly imagine future events in order to lead to greater valuation of future rewards and decreased valuation of brief intense stimuli that act as reinforcements of health-defeating behaviors [3]. Within the context of the pandemic, this may involve an awareness at managerial levels of the practical implications of public communication directed at stimulating personally relevant imagery of a future where all significant others are healthy. This suggests that engagement in brief intense stimuli such as use of public transportation, with resulting exposure to contagion, might be further reduced.

### Conclusion

It is concluded that students largely followed the voluntary recommendations implemented in Sweden during the onset of the COVID-19-pandemic; thus, coercive measures were not necessary. The present study found that compliance with public health recommendations among university students in Sweden was associated with observed symptoms of contagion and risk of contagion in the immediate vicinity. In relation to the efficiency of public health recommendations, the most important practical implication of our results is that health recommendations should include messages that stress the benefits of compliance, given the possibility that significant others may otherwise be at risk of contagion.

## Limitations

The questionnaire has not been validated and has limitations in relation to level of measurement, i.e., only nominal scales were used. A further limitation is that compliance with recommendations is not measured by using pre- and post-assessment of observed symptoms of contagion. In comparison to official figures presented by participating universities, women are somewhat overrepresented. It should be noted here that women generally are more willing to follow COVID-19 public health recommendations [7]. Other limitations are self-reports and a cross-sectional design. Although self-reports carry a risk of common method variance, with possible overestimation of associations [12], our analytic approach is conservative.

## Supplementary Information

**Additional file 1: Table S1.** Symptoms of contagion of cohabitants and self-reported recommendation compliance—contingency table.

**Additional file 2: Table S2.** Symptoms of contagion in cohabitants and self-reported recommendation compliance—analytic results.

**Additional file 3: Table S3.** Symptoms of contagion in family members and self-reported recommendation compliance—contingency table.

**Additional file 4: Table S4.** Symptoms of contagion in family members and self-reported recommendation compliance—analytic results.

**Additional file 5: Table S5.** Symptoms of contagion in circle of acquaintances and self-reported recommendation compliance—contingency table.

**Additional file 6: Table S6.** Symptoms of contagion in circle of acquaintances and self-reported recommendation compliance—analytic results.

**Additional file 7: Table S7.** Symptoms of contagion in someone else and self-reported recommendation compliance—contingency table.

**Additional file 8: Table S8.** Symptoms of contagion in someone else and self-reported recommendation compliance—analytic results.

**Additional file 9: Table S9.** Symptoms of contagion in another person and self-reported recommendation compliance—contingency table.

**Additional file 10: Table S10.** Symptoms of contagion in another person and self-reported recommendation compliance—analytic results.

**Additional file 11: Figure S1.** Compliance and self-reported symptoms of people living with the respondent.

**Additional file 12: Figure S2.** Compliance and self-reported symptoms of family members of the respondent (not living with).

**Additional file 13: Figure S3.** Compliance and self-reported symptoms of acquaintances of the respondent.

**Additional file 14: Figure S4.** Compliance and self-reported symptoms of others the respondent had contact with.

**Additional file 15: Figure S5.** Compliance and self-reported symptoms of another person.

**Additional file 16.** COVID-19 questions-general link national or international.

## Data Availability

The data generated or analyzed during the current study are available from the corresponding author on reasonable request.
