# Supplementary material for: Associations between compliance with covid-19 public health recommendations and perceived contagion in others: a self-report study in Swedish university students
Source: BMC Res Notes. 2021 Nov 25;14:429. doi: 10.1186/s13104-021-05848-6 (PMC8613723; doi:10.1186/s13104-021-05848-6)
Supplement: Supplementary file 1 — Additional file 1: Table S1. Symptoms of contagion of cohabitants and self-reported recommendation compliance—contingency table. [file 13104_2021_5848_MOESM1_ESM.docx]

Table S1. Symptoms of contagion of cohabitants and self-reported recommendation compliance – Contingency table.

| **Self-reported symptoms of someone the respondent lives with vs recommendation compliance** | | | | | | |
| --- | --- | --- | --- | --- | --- | --- |
|  | **No symptoms** | **Mild symptoms** | **Moderate symptoms** | **Severe symptoms** | **Died** | **Not relevant/Do not know** |
| **Handwashing with soap/alcohol** | | | | | | |
| **Compliance** | 2013 (96.4%) | 445 (94.7%) | 170 (95%) | 19 (95%) | 0 | 739 (94.6%) |
| **Non-compliance** | 75 (3.6%) | 25 (5.3%) | 9 (5%) | 1 (5%) | 0 | 42 (5.4%) |
| **Remained at home** | | | | | | |
| **Compliance** | 1739 (83.2%) | 382 (81.3%) | 138 (77.1%) | 15 (75%) | 0 | 619 (79.2%) |
| **Non-compliance** | 350 (16.8%) | 88 (18.7%) | 41 (22.9%) | 5 (25%) | 0 | 163 (20.8%) |
| **Sneezed/coughed in your arm** | | | | | | |
| **Compliance** | 1966 (94.5%) | 438 (93.2%) | 173 (96.6%) | 17 (85%) | 0 | 725 (92.7%) |
| **Non-compliance** | 115 (5.5%) | 32 (6.8%) | 6 (3.4%) | 3 (15%) | 0 | 57 (7.3%) |
| **Kept a distance from others when you have gone out** | | | | | | |
| **Compliance** | 1853 (88.7%) | 422 (89.8%) | 149 (83.2%) | 19 (95%) | 0 | 663 (84.8%) |
| **Non-compliance** | 237 (11.3%) | 48 (10.2%) | 30 (16.8%) | 1 (5%) | 0 | 119 (15.2%) |
| **Avoided meeting with persons who are older/in a risk group** | | | | | | |
| **Compliance** | 1995 (95.6%) | 454 (96.6%) | 174 (97.2%) | 20 (100%) | 0 | 753 (96.3%) |
| **Non-compliance** | 91 (4.4%) | 16 (3.4%) | 5 (2.8%) | 0 (0%) | 0 | 29 (3.7%) |
| **Avoided traveling with public transportation** | | | | | | |
| **Compliance** | 1511 (72.4%) | 314 (67%) | 127 (70.9%) | 8 (40%) | 0 | 511 (65.4%) |
| **Non-compliance** | 577 (27.6%) | 155 (33%) | 52 (29.1%) | 12 (60%) | 0 | 270 (34.6%) |
| **Avoided travel to other places in the country** | | | | | | |
| **Compliance** | 1807 (86.7%) | 401 (85.5%) | 160 (89.4%) | 17 (85%) | 0 | 688 (88.2%) |
| **Non-compliance** | 276 (13.3%) | 68 (14.5%) | 19 (10.6%) | 3 (15%) | 0 | 92 (11.8%) |
