# Supplementary material for: Associations between compliance with covid-19 public health recommendations and perceived contagion in others: a self-report study in Swedish university students
Source: BMC Res Notes. 2021 Nov 25;14:429. doi: 10.1186/s13104-021-05848-6 (PMC8613723; doi:10.1186/s13104-021-05848-6)
Supplement: Supplementary file 2 — Additional file 2: Table S2. Symptoms of contagion in cohabitants and self-reported recommendation compliance—analytic results. [file 13104_2021_5848_MOESM2_ESM.docx]

Table S2. Symptoms of contagion in cohabitants and self-reported recommendation compliance – analytic results.

|  | **Bayesian marginal posterior distribution** | | | | **Maximum likelihood estimates and null hypothesis testing** | |
| --- | --- | --- | --- | --- | --- | --- |
|  | **Normal priors** | | **Regularizing priors** | |  |  |
|  | **Median (2.5%; 97.5%)** | **OR > 1** | **Median (2.5%; 97.5%)** | **OR > 1** | **Estimate (95% CI)** | **p-value** |
| **Mild vs No symptoms** | | | | | | |
| **Age** | 1.00 (0.98; 1.01) | 49.3% | 1.00 (0.99; 1.00) | 47.3% | 1.01 (0.99; 1.03) | 0.447 |
| **Man vs Woman** | 0.88 (0.69; 0.95) | 13.8% | 1.00 (0.88; 1.00) | 43.0% | 0.89 (0.70; 1.12) | 0.320 |
| **Other vs Woman** | 1.38 (0.59; 1.80) | 77.7% | 1.00 (0.91; 1.01) | 52.1% | 1.53 (0.64; 3.66) | 0.335 |
| **Handwashing with soap/alcohol*** | 1.47 (0.92; 1.72) | 94.7% | 1.00 (0.94; 1.01) | 56.7% | 1.54 (0.96; 2.47) | 0.075 |
| **Remained at home*** | 1.07 (0.82; 1.17) | 69.1% | 1.00 (0.94; 1.01) | 53.2% | 1.08 (0.83; 1.42) | 0.566 |
| **Sneezed/coughed in your arm*** | 1.26 (0.82; 1.44) | 85.9% | 1.00 (0.94; 1.01) | 55.1% | 1.28 (0.85; 1.93) | 0.243 |
| **Kept a distance from others when you have gone out*** | 0.84 (0.60; 0.94) | 14.5% | 1.00 (0.81; 1.00) | 41.5% | 0.85 (0.61; 1.19) | 0.340 |
| **Avoided meeting with persons who are older/in a risk group*** | 0.75 (0.43; 0.90) | 13.9% | 1.00 (0.85; 1.00) | 46.5% | 0.73 (0.42; 1.26) | 0.260 |
| **Avoided traveling with public transportation*** | 1.24 (0.99; 1.35) | 96.7% | 1.00 (0.97; 1.03) | 65.2% | 1.27 (1.01; 1.61) | 0.040 |
| **Avoided travel to other places in the country*** | 1.07 (0.79; 1.19) | 68% | 1.00 (0.95; 1.01) | 57.5% | 1.09 (0.81; 1.47) | 0.581 |
| **Moderate vs No symptoms** | | | | | | |
| **Age** | 1.00 (0.97; 1.01) | 51.5% | 1.00 (0.98; 1.00) | 48.7% | 1.02 (0.99; 1.05) | 0.206 |
| **Man vs Woman** | 0.82 (0.56; 0.92) | 13.6% | 1.00 (0.82; 1.00) | 43.7% | 0.83 (0.58; 1.21) | 0.334 |
| **Other vs Woman** | 1.35 (0.40; 1.96) | 69.8% | 1.00 (0.87; 1.01) | 52.1% | 1.72 (0.50; 5.87) | 0.389 |
| **Handwashing with soap/alcohol*** | 1.37 (0.65; 1.73) | 80.8% | 1.00 (0.90; 1.01) | 52.0% | 1.51 (0.73; 3.10) | 0.266 |
| **Remained at home*** | 1.41 (0.95; 1.60) | 95.7% | 1.00 (0.95; 1.03) | 61.8% | 1.45 (0.99; 2.14) | 0.056 |
| **Sneezed/coughed in your arm*** | 0.62 (0.27; 0.80) | 9.7% | 1.00 (0.62; 1.00) | 42.4% | 0.60 (0.26; 1.38) | 0.228 |
| **Kept a distance from others when you have gone out*** | 1.55 (1.00; 1.79) | 97.7% | 1.00 (0.95; 1.03) | 62.3% | 1.64 (1.07; 2.53) | 0.024 |
| **Avoided meeting with persons who are older/in a risk group*** | 0.66 (0.27; 0.86) | 14.4% | 1.00 (0.75; 1.01) | 46.3% | 0.62 (0.25; 1.55) | 0.304 |
| **Avoided traveling with public transportation*** | 0.97 (0.68; 1.09) | 44% | 1.00 (0.89; 1.01) | 49.2% | 1.02 (0.71; 1.46) | 0.922 |
| **Avoided travel to other places in the country*** | 0.70 (0.42; 0.83) | 7.8% | 1.00 (0.74; 1.00) | 43.1% | 0.72 (0.43; 1.21) | 0.215 |
| **Severe vs No symptoms** | | | | | | |
| **Age** | 0.9 (0.84; 0.92) | 0% | 0.92 (0.86; 0.94) | 1.2% | 1.02 (0.94; 1.12) | 0.586 |
| **Man vs Woman** | 0.80 (0.31; 1.09) | 31.7% | 1.00 (0.66; 1.02) | 46.3% | 0.87 (0.31; 2.48) | 0.799 |
| **Other vs Woman** | 0.81 (0.13; 1.51) | 41.4% | 1.00 (0.40; 1.02) | 48.2% | NA^a^ | NA^a^ |
| **Handwashing with soap/alcohol*** | 1.00 (0.21; 1.59) | 50% | 1.00 (0.63; 1.03) | 50.0% | 1.16 (0.15; 9.18) | 0.886 |
| **Remained at home*** | 1.05 (0.39; 1.43) | 54.1% | 1.00 (0.75; 1.03) | 51.9% | 1.20 (0.42; 3.47) | 0.735 |
| **Sneezed/coughed in your arm*** | 2.10 (0.59; 3.12) | 88.4% | 1.00 (0.80; 1.06) | 58.2% | 3.53 (0.99; 12.66) | 0.053 |
| **Kept a distance from others when you have gone out*** | 0.45 (0.12; 0.68) | 9.1% | 1.00 (0.27; 1.01) | 39.8% | 0.32 (0.04; 2.41) | 0.266 |
| **Avoided meeting with persons who are older/in a risk group*** | 0.52 (0.10; 0.86) | 19.1% | 1.00 (0.16; 1.02) | 44.3% | NA^a^ | NA^a^ |
| **Avoided traveling with public transportation*** | 2.52 (1.11; 3.33) | 98.7% | 1.02 (0.91; 1.31) | 70.2% | 4.53 (1.73; 11.8) | 0.002 |
| **Avoided travel to other places in the country*** | 0.73 (0.24; 1.03) | 26.9% | 1.00 (0.65; 1.02) | 48.1% | 0.80 (0.22; 2.88) | 0.730 |
| **Not relevant/Do not know vs No symptoms** | | | | | | |
| **Age** | 1.02 (1.01; 1.03) | 99.7% | 1.02 (1.00; 1.03) | 99.3% | 1.03 (1.01; 1.05) | < 0.001 |
| **Man vs Woman** | 1.34 (1.12; 1.43) | 99.9% | 1.34 (1.07; 1.43) | 99.7% | 1.36 (1.13; 1.63) | 0.001 |
| **Other vs Woman** | 1.34 (0.63; 1.70) | 78.2% | 1.00 (0.80; 1.05) | 55.4% | 1.44 (0.67; 3.12) | 0.351 |
| **Handwashing with soap/alcohol*** | 1.32 (0.89; 1.50) | 91.6% | 1.01 (0.90; 1.08) | 65.3% | 1.35 (0.91; 2.01) | 0.135 |
| **Remained at home*** | 1.18 (0.95; 1.27) | 93.3% | 1.03 (0.95; 1.10) | 74.9% | 1.19 (0.96; 1.48) | 0.114 |
| **Sneezed/coughed in your arm*** | 1.17 (0.83; 1.32) | 82% | 1.01 (0.89; 1.06) | 62.6% | 1.18 (0.84; 1.67) | 0.333 |
| **Kept a distance from others when you have gone out*** | 1.28 (1.01; 1.40) | 97.8% | 1.11 (0.97; 1.24) | 86.3% | 1.30 (1.01; 1.67) | 0.038 |
| **Avoided meeting with persons who are older/in a risk group*** | 0.76 (0.49; 0.88) | 9.6% | 0.99 (0.72; 1.01) | 38.5% | 0.74 (0.48; 1.14) | 0.175 |
| **Avoided traveling with public transportation*** | 1.38 (1.14; 1.47) | >99.9% | 1.25 (1.00; 1.34) | 97.5% | 1.41 (1.17; 1.70) | < 0.001 |
| **Avoided travel to other places in the country*** | 0.78 (0.60; 0.85) | 3% | 0.96 (0.72; 1.00) | 23.3% | 0.78 (0.60; 1.02) | 0.073 |
| * Non-compliant vs Compliant ^a^ Not estimable in the MLE model due to zero entries | | | | | | |
