# Supplementary material for: Associations between compliance with covid-19 public health recommendations and perceived contagion in others: a self-report study in Swedish university students
Source: BMC Res Notes. 2021 Nov 25;14:429. doi: 10.1186/s13104-021-05848-6 (PMC8613723; doi:10.1186/s13104-021-05848-6)
Supplement: Supplementary file 3 — Additional file 3: Table S3. Symptoms of contagion in family members and self-reported recommendation compliance—contingency table. [file 13104_2021_5848_MOESM3_ESM.docx]

Table S3. Symptoms of contagion in family members and self-reported recommendation compliance – Contingency table.

| **Self-reported symptoms of someone in the respondent’s family that they do not live with vs recommendation compliance** | | | | | | |
| --- | --- | --- | --- | --- | --- | --- |
|  | **No symptoms** | **Mild symptoms** | **Moderate symptoms** | **Severe symptoms** | **Died** | **Not relevant/Do not know** |
| **Handwashing with soap/alcohol** | | | | | | |
| **Compliance** | 2041 (95.8%) | 435 (96.7%) | 329 (96.8%) | 81 (97.6%) | 20 (90.9%) | 501 (94.2%) |
| **Non-compliance** | 89 (4.2%) | 15 (3.3%) | 11 (3.2%) | 2 (2.4%) | 2 (9.1%) | 31 (5.8%) |
| **Remained at home** | | | | | | |
| **Compliance** | 1756 (82.4%) | 361 (80.4%) | 280 (82.4%) | 69 (83.1%) | 16 (72.7%) | 428 (80.5%) |
| **Non-compliance** | 376 (17.6%) | 88 (19.6%) | 60 (17.6%) | 14 (16.9%) | 6 (27.3%) | 104 (19.5%) |
| **Sneezed/coughed in your arm** | | | | | | |
| **Compliance** | 1994 (93.8%) | 433 (96.2%) | 320 (94.4%) | 78 (94%) | 21 (95.5%) | 493 (92.7%) |
| **Non-compliance** | 131 (6.2%) | 17 (3.8%) | 19 (5.6%) | 5 (6%) | 1 (4.5%) | 39 (7.3%) |
| **Kept a distance from others when you have gone out** | | | | | | |
| **Compliance** | 1875 (87.9%) | 407 (90.4%) | 303 (89.1%) | 70 (84.3%) | 18 (81.8%) | 448 (84.2%) |
| **Non-compliance** | 258 (12.1%) | 43 (9.6%) | 37 (10.9%) | 13 (15.7%) | 4 (18.2%) | 84 (15.8%) |
| **Avoided meeting with persons who are older/in a risk group** | | | | | | |
| **Compliance** | 2039 (95.7%) | 440 (97.8%) | 330 (97.1%) | 79 (95.2%) | 19 (86.4%) | 508 (95.5%) |
| **Non-compliance** | 91 (4.3%) | 10 (2.2%) | 10 (2.9%) | 4 (4.8%) | 3 (13.6%) | 24 (4.5%) |
| **Avoided traveling with public transportation** | | | | | | |
| **Compliance** | 1522 (71.4%) | 313 (69.6%) | 231 (68.1%) | 54 (65.1%) | 13 (59.1%) | 360 (67.8%) |
| **Non-compliance** | 610 (28.6%) | 137 (30.4%) | 108 (31.9%) | 29 (34.9%) | 9 (40.9%) | 171 (32.2%) |
| **Avoided travel to other places in the country** | | | | | | |
| **Compliance** | 1838 (86.4%) | 396 (88%) | 301 (89.1%) | 71 (85.5%) | 17 (81%) | 466 (87.9%) |
| **Non-compliance** | 289 (13.6%) | 54 (12%) | 37 (10.9%) | 12 (14.5%) | 4 (19%) | 64 (12.1%) |
