# Supplementary material for: Associations between compliance with covid-19 public health recommendations and perceived contagion in others: a self-report study in Swedish university students
Source: BMC Res Notes. 2021 Nov 25;14:429. doi: 10.1186/s13104-021-05848-6 (PMC8613723; doi:10.1186/s13104-021-05848-6)
Supplement: Supplementary file 4 — Additional file 4: Table S4. Symptoms of contagion in family members and self-reported recommendation compliance—analytic results. [file 13104_2021_5848_MOESM4_ESM.docx]

Table S4. Symptoms of contagion in family members and self-reported recommendation compliance – analytic results.

|  | **Bayesian marginal posterior distribution** | | | | | **Maximum likelihood estimates and null hypothesis testing** | | | | |
| --- | --- | --- | --- | --- | --- | --- | --- | --- | --- | --- |
|  | **Normal priors** | | **Regularizing priors** | | |  | |  | | |
|  | **Median (2.5%; 97.5%)** | **OR > 1** | **Median (2.5%; 97.5%)** | | **OR > 1** | **Estimate (95% CI)** | | **p-value** | | |
| **Mild vs No symptoms** | | | | | | | | | |  |
| **Age** | 0.98 (0.96; 0.99) | 4% | 0.99 (0.97; 1.00) | 12.5% | | | 0.99 (0.97; 1.01) | | 0.343 |  |
| **Man vs Woman** | 0.72 (0.56; 0.78) | 0.3% | 0.76 (0.57; 0.87) | 5% | | | 0.72 (0.56; 0.93) | | 0.010 |  |
| **Other vs Woman** | 1.19 (0.48; 1.57) | 65.7% | 1.00 (0.81; 1.03) | 52.4% | | | 1.21 (0.49; 3.02) | | 0.680 |  |
| **Handwashing with soap/alcohol*** | 0.85 (0.48; 1.03) | 28.9% | 1.00 (0.72; 1.01) | 42.5% | | | 0.86 (0.48; 1.54) | | 0.617 |  |
| **Remained at home*** | 1.15 (0.87; 1.27) | 84.9% | 1.00 (0.93; 1.04) | 61.7% | | | 1.17 (0.89; 1.53) | | 0.266 |  |
| **Sneezed/coughed in your arm*** | 0.66 (0.38; 0.78) | 4.6% | 0.98 (0.52; 1.00) | 28.4% | | | 0.65 (0.38; 1.11) | | 0.111 |  |
| **Kept a distance from others when you have gone out*** | 0.77 (0.54; 0.86) | 6.3% | 0.98 (0.66; 1.00) | 28.7% | | | 0.78 (0.55; 1.11) | | 0.161 |  |
| **Avoided meeting with persons who are older/in a risk group*** | 0.58 (0.30; 0.71) | 3.6% | 0.99 (0.47; 1.00) | 31.4% | | | 0.56 (0.29; 1.09) | | 0.090 |  |
| **Avoided traveling with public transportation*** | 1.11 (0.87; 1.20) | 80.1% | 1.00 (0.91; 1.02) | 53.7% | | | 1.13 (0.89; 1.44) | | 0.312 |  |
| **Avoided travel to other places in the country*** | 0.83 (0.59; 0.93) | 14% | 1.00 (0.78; 1.01) | 39.4% | | | 0.85 (0.61; 1.18) | | 0.323 |  |
| **Moderate vs No symptoms** | | | | | | | | | |  |
| **Age** | 0.99 (0.97; 0.99) | 12.8% | 1.00 (0.97; 1.00) | 24.6% | | | 1.00 (0.97; 1.02) | | 0.788 |  |
| **Man vs Woman** | 0.52 (0.38; 0.57) | < 0.01% | 0.54 (0.39; 0.61) | < 0.01% | | | 0.52 (0.38; 0.7) | | < 0.001 |  |
| **Other vs Woman** | 0.43 (0.11; 0.64) | 7.1% | 1.00 (0.26; 1.01) | 39.1% | | | 0.24 (0.03; 1.83) | | 0.170 |  |
| **Handwashing with soap/alcohol*** | 0.91 (0.47; 1.12) | 38.3% | 1.00 (0.79; 1.01) | 47.5% | | | 0.93 (0.49; 1.77) | | 0.821 |  |
| **Remained at home*** | 0.99 (0.72; 1.1) | 46.4% | 1.00 (0.88; 1.01) | 48.6% | | | 1.00 (0.73; 1.36) | | 0.981 |  |
| **Sneezed/coughed in your arm*** | 1.06 (0.63; 1.26) | 59.9% | 1.00 (0.86; 1.02) | 52.3% | | | 1.08 (0.65; 1.78) | | 0.768 |  |
| **Kept a distance from others when you have gone out*** | 0.92 (0.62; 1.04) | 32% | 1.00 (0.83; 1.01) | 45.5% | | | 0.94 (0.64; 1.37) | | 0.743 |  |
| **Avoided meeting with persons who are older/in a risk group*** | 0.74 (0.37; 0.91) | 16.6% | 1.00 (0.72; 1.01) | 43.5% | | | 0.74 (0.38; 1.44) | | 0.370 |  |
| **Avoided traveling with public transportation*** | 1.23 (0.95; 1.35) | 93.9% | 1.00 (0.94; 1.04) | 62.2% | | | 1.27 (0.97; 1.65) | | 0.082 |  |
| **Avoided travel to other places in the country*** | 0.80 (0.54; 0.9) | 11.2% | 1.00 (0.78; 1.01) | 41.0% | | | 0.81 (0.55; 1.18) | | 0.263 |  |
| **Severe vs No symptoms** | | | | | | | | | |  |
| **Age** | 0.98 (0.95; 1.00) | 18.3% | 1.00 (0.96; 1.00) | 33.1% | | | 1.02 (0.98; 1.07) | | 0.281 |  |
| **Man vs Woman** | 0.93 (0.57; 1.09) | 37.6% | 1.00 (0.88; 1.01) | 48.8% | | | 0.97 (0.59; 1.60) | | 0.914 |  |
| **Other vs Woman** | 0.56 (0.10; 0.95) | 23.1% | 1.00 (0.65; 1.01) | 48.1% | | | NA^a^ | | NA^a^ |  |
| **Handwashing with soap/alcohol*** | 0.61 (0.18; 0.88) | 17.5% | 1.00 (0.74; 1.01) | 47.1% | | | 0.54 (0.13; 2.25) | | 0.396 |  |
| **Remained at home*** | 0.82 (0.45; 1.00) | 24.8% | 1.00 (0.84; 1.01) | 47.3% | | | 0.84 (0.46; 1.55) | | 0.581 |  |
| **Sneezed/coughed in your arm*** | 0.95 (0.37; 1.25) | 45.2% | 1.00 (0.85; 1.01) | 50.2% | | | 0.98 (0.38; 2.49) | | 0.963 |  |
| **Kept a distance from others when you have gone out*** | 1.19 (0.64; 1.46) | 71.8% | 1.00 (0.91; 1.01) | 53.1% | | | 1.33 (0.71; 2.5) | | 0.368 |  |
| **Avoided meeting with persons who are older/in a risk group*** | 1.02 (0.37; 1.39) | 51.8% | 1.00 (0.87; 1.01) | 50.7% | | | 1.06  (0.38; 3.01) | | 0.906 |  |
| **Avoided traveling with public transportation*** | 1.25 (0.76; 1.46) | 81.8% | 1.00 (0.92; 1.01) | 53.3% | | | 1.40 (0.85; 2.29) | | 0.183 |  |
| **Avoided travel to other places in the country*** | 0.95 (0.49; 1.16) | 43.5% | 1.00 (0.89; 1.01) | 51.3% | | | 1.03 (0.54; 1.97) | | 0.934 |  |
| **Died vs No Symptoms** | | | | | | | | | |  |
| **Age** | 0.89 (0.83; 0.91) | 0% | 0.91  (0.85; 0.93) | 0.5% | | | 1.00 (0.91; 1.09) | | 0.953 |  |
| **Man vs Woman** | 0.95 (0.4; 1.26) | 45.3% | 1.00 (0.76; 1.02) | 50.3% | | | 1.05 (0.41; 2.71) | | 0.919 |  |
| **Other vs Woman** | 0.82 (0.13; 1.48) | 41% | 1.00 (0.47; 1.02) | 48.1% | | | NA^a^ | | NA^a^ |  |
| **Handwashing with soap/alcohol*** | 1.40 (0.36; 2.14) | 69.7% | 1.00 (0.78; 1.03) | 54.1% | | | 2.08 (0.45; 9.54) | | 0.346 |  |
| **Remained at home*** | 1.30 (0.51; 1.74) | 72.3% | 1.00 (0.83; 1.04) | 55.3% | | | 1.52 (0.56; 4.10) | | 0.414 |  |
| **Sneezed/coughed in your arm*** | 0.77 (0.17; 1.21) | 35% | 1.00 (0.64; 1.02) | 48.4% | | | 0.59 (0.08; 4.66) | | 0.619 |  |
| **Kept a distance from others when you have gone out*** | 1.07 (0.37; 1.50) | 55.1% | 1.00 (0.79; 1.03) | 53.0% | | | 1.27 (0.40; 3.99) | | 0.688 |  |
| **Avoided meeting with persons who are older/in a risk group*** | 2.14 (0.58; 3.17) | 88.7% | 1.00 (0.83; 1.06) | 58.3% | | | 3.35 (0.93; 12.01) | | 0.063 |  |
| **Avoided traveling with public transportation*** | 1.21 (0.52; 1.6) | 67.1% | 1.00 (0.82; 1.03) | 54.3% | | | 1.51 (0.59; 3.87) | | 0.386 |  |
| **Avoided travel to other places in the country*** | 0.92 (0.32; 1.28) | 43.7% | 1.00 (0.77; 1.03) | 52.3% | | | 1.06 (0.33; 3.4) | | 0.923 |  |
| **Not relevant/do not know vs No symptoms** | | | | | | | | | |  |
| **Age** | 1.05 (1.03; 1.06) | >99.9% | 1.05 (1.03; 1.06) | >99.9% | | | 1.06 (1.04; 1.08) | | < 0.001 |  |
| **Man vs Woman** | 1.16 (0.94; 1.25) | 92% | 1.03 (0.96; 1.12) | 77.9% | | | 1.18 (0.95; 1.46) | | 0.129 |  |
| **Other vs Woman** | 1.8 (0.84; 2.32) | 93.8% | 1.01 (0.88; 1.1) | 64.5% | | | 1.88 (0.88; 4.05) | | 0.106 |  |
| **Handwashing with soap/alcohol*** | 1.33 (0.86; 1.53) | 90.1% | 1.02 (0.93; 1.12) | 69.2% | | | 1.35 (0.88; 2.08) | | 0.173 |  |
| **Remained at home*** | 1.06 (0.82; 1.15) | 66.6% | 1.00 (0.92; 1.04) | 60.6% | | | 1.07 (0.83; 1.38) | | 0.619 |  |
| **Sneezed/coughed in your arm*** | 1.05 (0.71; 1.2) | 60.5% | 1.00 (0.89; 1.04) | 57.0% | | | 1.04 (0.71; 1.53) | | 0.831 |  |
| **Kept a distance from others when you have gone out*** | 1.35 (1.02; 1.5) | 98.3% | 1.18 (0.98; 1.36) | 88.3% | | | 1.39 (1.05; 1.84) | | 0.020 |  |
| **Avoided meeting with persons who are older/in a risk group*** | 0.93 (0.58; 1.09) | 37.7% | 1.00 (0.84; 1.02) | 51.8% | | | 0.93 (0.58; 1.49) | | 0.758 |  |
| **Avoided traveling with public transportation*** | 1.24 (0.99; 1.34) | 97.2% | 1.03 (0.96; 1.11) | 77.5% | | | 1.28 (1.03; 1.6) | | 0.029 |  |
| **Avoided travel to other places in the country*** | 0.86 (0.63; 0.95) | 15.2% | 1.00 (0.84; 1.01) | 44.8% | | | 0.87 (0.64; 1.18) | | 0.374 |  |
| * Non-compliant vs Compliant ^a^ Not estimable in the MLE model due to zero entries | | | | | | | | | |  |
