# Supplementary material for: Associations between compliance with covid-19 public health recommendations and perceived contagion in others: a self-report study in Swedish university students
Source: BMC Res Notes. 2021 Nov 25;14:429. doi: 10.1186/s13104-021-05848-6 (PMC8613723; doi:10.1186/s13104-021-05848-6)
Supplement: Supplementary file 5 — Additional file 5: Table S5. Symptoms of contagion in circle of acquaintances and self-reported recommendation compliance—contingency table. [file 13104_2021_5848_MOESM5_ESM.docx]

Table S5. Symptoms of contagion in circle of acquaintances and self-reported recommendation compliance – Contingency table.

| **Self-reported symptoms of acquaintances vs recommendation compliance** | | | | | | |
| --- | --- | --- | --- | --- | --- | --- |
|  | **No symptoms** | **Mild symptoms** | **Moderate symptoms** | **Severe symptoms** | **Died** | **Not relevant/Do not know** |
| **Handwashing with soap/alcohol** | | | | | | |
| **Compliance** | 1217 (96.3%) | 691 (95.3%) | 856 (96%) | 227 (96.2%) | 59 (98.3%) | 405 (94.4%) |
| **Non-compliance** | 47 (3.7%) | 34 (4.7%) | 36 (4%) | 9 (3.8%) | 1 (1.7%) | 24 (5.6%) |
| **Remained at home** | | | | | | |
| **Compliance** | 1060 (83.8%) | 583 (80.4%) | 710 (79.6%) | 179 (75.8%) | 52 (86.7%) | 365 (84.9%) |
| **Non-compliance** | 205 (16.2%) | 142 (19.6%) | 182 (20.4%) | 57 (24.2%) | 8 (13.3%) | 65 (15.1%) |
| **Sneezed/coughed in your arm** | | | | | | |
| **Compliance** | 1183 (93.9%) | 681 (94.1%) | 847 (95%) | 216 (91.9%) | 59 (98.3%) | 397 (92.5%) |
| **Non-compliance** | 77 (6.1%) | 43 (5.9%) | 45 (5%) | 19 (8.1%) | 1 (1.7%) | 32 (7.5%) |
| **Kept a distance from others when you have gone out** | | | | | | |
| **Compliance** | 1120 (88.5%) | 631 (87.2%) | 777 (87.1%) | 211 (89.4%) | 57 (95%) | 369 (85.8%) |
| **Non-compliance** | 146 (11.5%) | 93 (12.8%) | 115 (12.9%) | 25 (10.6%) | 3 (5%) | 61 (14.2%) |
| **Avoided meeting with persons who are older/in a risk group** | | | | | | |
| **Compliance** | 1212 (96%) | 696 (96%) | 868 (97.3%) | 225 (95.3%) | 58 (96.7%) | 402 (93.5%) |
| **Non-compliance** | 51 (4%) | 29 (4%) | 24 (2.7%) | 11 (4.7%) | 2 (3.3%) | 28 (6.5%) |
| **Avoided traveling with public transportation** | | | | | | |
| **Compliance** | 923 (73%) | 496 (68.6%) | 600 (67.3%) | 161 (68.2%) | 43 (71.7%) | 296 (68.8%) |
| **Non-compliance** | 342 (27%) | 227 (31.4%) | 291 (32.7%) | 75 (31.8%) | 17 (28.3%) | 134 (31.2%) |
| **Avoided travel to other places in the country** | | | | | | |
| **Compliance** | 1094 (86.6%) | 630 (87.1%) | 779 (87.7%) | 202 (86%) | 52 (86.7%) | 376 (87.6%) |
| **Non-compliance** | 169 (13.4%) | 93 (12.9%) | 109 (12.3%) | 33 (14%) | 8 (13.3%) | 53 (12.4%) |
