# Supplementary material for: Associations between compliance with covid-19 public health recommendations and perceived contagion in others: a self-report study in Swedish university students
Source: BMC Res Notes. 2021 Nov 25;14:429. doi: 10.1186/s13104-021-05848-6 (PMC8613723; doi:10.1186/s13104-021-05848-6)
Supplement: Supplementary file 6 — Additional file 6: Table S6. Symptoms of contagion in circle of acquaintances and self-reported recommendation compliance—analytic results. [file 13104_2021_5848_MOESM6_ESM.docx]

Table S6. Symptoms of contagion in circle of acquaintances and self-reported recommendation compliance – Analytic results.

|  | **Bayesian marginal posterior distribution** | | | | **Maximum likelihood estimates and null hypothesis testing** | |
| --- | --- | --- | --- | --- | --- | --- |
|  | **Normal priors** | | **Regularizing priors** | |  |  |
|  | **Median (2.5%; 97.5%)** | **OR > 1** | **Median (2.5%; 97.5%)** | **OR > 1** | **Estimate (95% CI)** | **p-value** |
| Mild vs No symptoms | | | | | | |
| Age | 0.97 (0.95; 0.98) | 0.1% | 0.97 (0.95; 0.98) | 0.1% | 0.98 (0.96; 1) | 0.017 |
| Man vs **Woman** | 0.78 (0.63; 0.84) | 1.1% | 0.99 (0.78; 1) | 30.5% | 0.78 (0.63; 0.97) | 0.025 |
| Other vs **Woman** | 0.92 (0.4; 1.19) | 41.4% | 1.00 (0.84; 1.01) | 49.7% | 0.90 (0.38; 2.14) | 0.815 |
| Handwashing with soap/alcohol* | 1.32 (0.84; 1.53) | 88.8% | 1.00 (0.92; 1.02) | 55.6% | 1.35 (0.85; 2.13) | 0.204 |
| Remained at home* | 1.18 (0.93; 1.28) | 90.8% | 1.00 (0.95; 1.02) | 59.9% | 1.19 (0.93; 1.52) | 0.175 |
| Sneezed/coughed in your arm* | 1.04 (0.71; 1.19) | 58.3% | 1.00 (0.90; 1.01) | 51.3% | 1.03 (0.70; 1.53) | 0.866 |
| Kept a distance from others when you have gone out* | 1.08 (0.81; 1.19) | 70.0% | 1.00 (0.92; 1.01) | 52.4% | 1.09 (0.82; 1.45) | 0.568 |
| Avoided meeting with persons who are older/in a risk group* | 1.01 (0.63; 1.18) | 51.3% | 1.00 (0.89; 1.01) | 51.1% | 1.01 (0.63; 1.62) | 0.975 |
| Avoided traveling with public transportation* | 1.14 (0.92; 1.23) | 88.4% | 1.00 (0.94; 1.01) | 54.5% | 1.16 (0.94; 1.44) | 0.174 |
| Avoided travel to other places in the country* | 0.88 (0.67; 0.97) | 18.6% | 1.00 (0.89; 1.01) | 46.4% | 0.89 (0.67; 1.18) | 0.412 |
| Moderate vs No symptoms | | | | | | |
| Age | 1.01 (1.00; 1.02) | 92% | 1.00 (0.99; 1.01) | 74.8% | 1.02 (1; 1.04) | 0.026 |
| Man vs **Woman** | 0.78 (0.64; 0.84) | 0.8% | 0.98 (0.76; 1.00) | 24.8% | 0.79 (0.64; 0.96) | 0.019 |
| Other vs **Woman** | 0.74 (0.32; 0.97) | 22.4% | 1.00 (0.72; 1.01) | 44.5% | 0.71 (0.3; 1.68) | 0.431 |
| Handwashing with soap/alcohol* | 1.16 (0.74; 1.34) | 74.7% | 1.00 (0.87; 1.01) | 50.1% | 1.18 (0.75; 1.85) | 0.48 |
| Remained at home* | 1.24 (0.99; 1.34) | 96.7% | 1.01 (0.97; 1.08) | 72.5% | 1.25 (0.99; 1.57) | 0.061 |
| Sneezed/coughed in your arm* | 0.85 (0.57; 0.97) | 20.2% | 1.00 (0.76; 1.00) | 37.9% | 0.84 (0.57; 1.24) | 0.381 |
| Kept a distance from others when you have gone out* | 1.15 (0.88; 1.26) | 83.9% | 1.00 (0.94; 1.02) | 58.6% | 1.16 (0.88; 1.52) | 0.292 |
| Avoided meeting with persons who are older/in a risk group* | 0.66 (0.40; 0.78) | 4.5% | 0.99 (0.53; 1.00) | 29.2% | 0.65 (0.40; 1.08) | 0.094 |
| Avoided traveling with public transportation* | 1.31 (1.07; 1.40) | 99.5% | 1.03 (0.98; 1.13) | 79.1% | 1.34 (1.10; 1.64) | 0.004 |
| Avoided travel to other places in the country* | 0.84 (0.64; 0.93) | 10.9% | 1.00 (0.85; 1.00) | 41.0% | 0.85 (0.65; 1.12) | 0.259 |
| Severe vs No symptoms | | | | | | |
| Age | 1.02 (1.00; 1.03) | 96.2% | 1.01 (0.99; 1.02) | 83.3% | 1.04 (1.01; 1.07) | 0.004 |
| Man vs **Woman** | 0.86 (0.63; 0.96) | 18.2% | 1.00 (0.88; 1.01) | 47.9% | 0.88 (0.64; 1.22) | 0.455 |
| Other vs **Woman** | 1.21 (0.41; 1.69) | 64.8% | 1.00 (0.87; 1.01) | 52.4% | 1.31 (0.43; 4.02) | 0.639 |
| Handwashing with soap/alcohol* | 0.99 (0.47; 1.26) | 49.3% | 1.00 (0.84; 1.01) | 48.7% | 1.03 (0.49; 2.16) | 0.935 |
| Remained at home* | 1.53 (1.08; 1.72) | 99.2% | 1.01 (0.96; 1.11) | 69.0% | 1.57 (1.10; 2.23) | 0.012 |
| Sneezed/coughed in your arm* | 1.32 (0.76; 1.58) | 84.7% | 1.00 (0.93; 1.02) | 56.9% | 1.33 (0.78; 2.27) | 0.29 |
| Kept a distance from others when you have gone out* | 0.85 (0.54; 0.99) | 24.1% | 1.00 (0.82; 1.01) | 44.8% | 0.87 (0.55; 1.39) | 0.564 |
| Avoided meeting with persons who are older/in a risk group* | 1.05 (0.54; 1.32) | 56.6% | 1.00 (0.89; 1.01) | 52.1% | 1.07 (0.54; 2.12) | 0.839 |
| Avoided traveling with public transportation* | 1.17 (0.84; 1.3) | 82.6% | 1.00 (0.93; 1.01) | 54.7% | 1.22 (0.88; 1.69) | 0.228 |
| Avoided travel to other places in the country* | 1.01 (0.66; 1.17) | 52.6% | 1.00 (0.92; 1.01) | 54.2% | 1.05 (0.69; 1.6) | 0.819 |
| Died vs No Symptoms | | | | | | |
| Age | 0.99 (0.95; 1) | 32% | 1.00 (0.96; 1) | 39.3% | 1.04 (0.99; 1.09) | 0.103 |
| Man vs **Woman** | 0.78 (0.42; 0.95) | 20% | 1.00 (0.79; 1.01) | 45.6% | 0.85 (0.46; 1.58) | 0.603 |
| Other vs **Woman** | 0.58 (0.11; 0.98) | 24.5% | 1.00 (0.64; 1.01) | 48.1% | NA^a^ | NA^a^ |
| Handwashing with soap/alcohol* | 0.63 (0.14; 0.98) | 24.3% | 1.00 (0.68; 1.01) | 47.1% | 0.52 (0.07; 3.90) | 0.527 |
| Remained at home* | 0.77 (0.36; 0.99) | 24.4% | 1.00 (0.71; 1.00) | 44.4% | 0.80 (0.37; 1.76) | 0.579 |
| Sneezed/coughed in your arm* | 0.45 (0.12; 0.67) | 8.3% | 1.00 (0.47; 1.00) | 43.9% | 0.27 (0.04; 2.01) | 0.202 |
| Kept a distance from others when you have gone out* | 0.48 (0.17; 0.66) | 5.4% | 1.00 (0.39; 1.00) | 39.9% | 0.44 (0.13; 1.46) | 0.182 |
| Avoided meeting with persons who are older/in a risk group* | 0.88 (0.25; 1.29) | 41.1% | 1.00 (0.80; 1.01) | 49.6% | 0.93 (0.22; 3.96) | 0.922 |
| Avoided traveling with public transportation* | 1.09 (0.60; 1.32) | 61.1% | 1.00 (0.84; 1.01) | 48.4% | 1.26 (0.68; 2.33) | 0.458 |
| Avoided travel to other places in the country* | 1.03 (0.47; 1.32) | 52.8% | 1.00 (0.86; 1.01) | 49.8% | 1.20 (0.54; 2.66) | 0.654 |
| **Not relevant/do not know vs No symptoms** | | | | | | |
| Age | 1.03 (1.01; 1.04) | >99.9% | 1.03 (1.01; 1.03) | 99.5% | 1.05 (1.02; 1.07) | < 0.001 |
| Man vs **Woman** | 1.23 (0.97; 1.34) | 95.8% | 1.26 (0.99; 1.40) | 94.3% | 1.25 (0.98; 1.60) | 0.069 |
| Other vs **Woman** | 1.38 (0.57; 1.83) | 76.9% | 1.00 (0.83; 1.05) | 57.1% | 1.46 (0.59; 3.62) | 0.419 |
| Handwashing with soap/alcohol* | 1.39 (0.84; 1.64) | 89.8% | 1.01 (0.90; 1.07) | 63.2% | 1.43 (0.85; 2.40) | 0.173 |
| Remained at home* | 0.81 (0.60; 0.90) | 9.2% | 0.97 (0.67; 1.00) | 23.9% | 0.82 (0.60; 1.12) | 0.215 |
| Sneezed/coughed in your arm* | 1.01 (0.65; 1.18) | 52.6% | 1.00 (0.86; 1.03) | 54.3% | 1.00 (0.64; 1.57) | 0.985 |
| Kept a distance from others when you have gone out* | 1.23 (0.88; 1.37) | 88.6% | 1.01 (0.93; 1.08) | 67.1% | 1.25 (0.90; 1.75) | 0.188 |
| Avoided meeting with persons who are older/in a risk group* | 1.52 (0.94; 1.79) | 95.6% | 1.08 (0.95; 1.36) | 78.4% | 1.54 (0.95; 2.51) | 0.081 |
| Avoided traveling with public transportation* | 1.31 (1.01; 1.43) | 97.9% | 1.01 (0.94; 1.07) | 69.7% | 1.35 (1.05; 1.75) | 0.021 |
| Avoided travel to other places in the country* | 0.87 (0.61; 0.97) | 20.4% | 1.00 (0.84; 1.02) | 48.2% | 0.88 (0.62; 1.25) | 0.488 |
| * Non-compliant vs Compliant ^a^ Not estimable in the MLE model due to zero entries | | | | | | |
