# Supplementary material for: Associations between compliance with covid-19 public health recommendations and perceived contagion in others: a self-report study in Swedish university students
Source: BMC Res Notes. 2021 Nov 25;14:429. doi: 10.1186/s13104-021-05848-6 (PMC8613723; doi:10.1186/s13104-021-05848-6)
Supplement: Supplementary file 7 — Additional file 7: Table S7. Symptoms of contagion in someone else and self-reported recommendation compliance—contingency table. [file 13104_2021_5848_MOESM7_ESM.docx]

Table S7. Symptoms of contagion in someone else and self-reported recommendation compliance – Contingency table.

| **Self-reported symptoms of others the respondent had contact with vs recommendation compliance** | | | | | | |
| --- | --- | --- | --- | --- | --- | --- |
|  | **No symptoms** | **Mild symptoms** | **Moderate symptoms** | **Severe symptoms** | **Died** | **Not relevant/Do not know** |
| **Handwashing with soap/alcohol** | | | | | | |
| **Compliance** | 1509 (95.7%) | 355 (94.7%) | 313 (96%) | 164 (98.8%) | 71 (97.3%) | 967 (95.9%) |
| **Non-compliance** | 68 (4.3%) | 20 (5.3%) | 13 (4%) | 2 (1.2%) | 2 (2.7%) | 41 (4.1%) |
| **Remained at home** | | | | | | |
| **Compliance** | 1320 (83.7%) | 294 (78.4%) | 240 (73.6%) | 125 (75.3%) | 60 (82.2%) | 841 (83.4%) |
| **Non-compliance** | 258 (16.3%) | 81 (21.6%) | 86 (26.4%) | 41 (24.7%) | 13 (17.8%) | 167 (16.6%) |
| **Sneezed/coughed in your arm** | | | | | | |
| **Compliance** | 1490 (94.6%) | 349 (93.1%) | 306 (94.2%) | 156 (94%) | 67 (93.1%) | 943 (93.6%) |
| **Non-compliance** | 85 (5.4%) | 26 (6.9%) | 19 (5.8%) | 10 (6%) | 5 (6.9%) | 64 (6.4%) |
| **Kept a distance from others when you have gone out** | | | | | | |
| **Compliance** | 1399 (88.6%) | 320 (85.3%) | 289 (88.7%) | 150 (90.4%) | 68 (93.2%) | 869 (86.2%) |
| **Non-compliance** | 180 (11.4%) | 55 (14.7%) | 37 (11.3%) | 16 (9.6%) | 5 (6.8%) | 139 (13.8%) |
| **Avoided meeting with persons who are older/in a risk group** | | | | | | |
| **Compliance** | 1510 (95.8%) | 367 (97.9%) | 314 (96.3%) | 157 (94.6%) | 69 (94.5%) | 965 (95.7%) |
| **Non-compliance** | 67 (4.2%) | 8 (2.1%) | 12 (3.7%) | 9 (5.4%) | 4 (5.5%) | 43 (4.3%) |
| **Avoided traveling with public transportation** | | | | | | |
| **Compliance** | 1143 (72.4%) | 251 (66.9%) | 221 (67.8%) | 108 (65.1%) | 60 (82.2%) | 691 (68.6%) |
| **Non-compliance** | 435 (27.6%) | 124 (33.1%) | 105 (32.2%) | 58 (34.9%) | 13 (17.8%) | 316 (31.4%) |
| **Avoided travel to other places in the country** | | | | | | |
| **Compliance** | 1362 (86.5%) | 331 (88.3%) | 285 (87.7%) | 136 (81.9%) | 66 (91.7%) | 884 (88.1%) |
| **Non-compliance** | 213 (13.5%) | 44 (11.7%) | 40 (12.3%) | 30 (18.1%) | 6 (8.3%) | 119 (11.9%) |
