# Supplementary material for: Associations between compliance with covid-19 public health recommendations and perceived contagion in others: a self-report study in Swedish university students
Source: BMC Res Notes. 2021 Nov 25;14:429. doi: 10.1186/s13104-021-05848-6 (PMC8613723; doi:10.1186/s13104-021-05848-6)
Supplement: Supplementary file 8 — Additional file 8: Table S8. Symptoms of contagion in someone else and self-reported recommendation compliance—analytic results. [file 13104_2021_5848_MOESM8_ESM.docx]

Table S8. Symptoms of contagion in someone else and self-reported recommendation compliance – Analytic results.

|  | **Bayesian marginal posterior distribution** | | | | **Maximum likelihood estimates and null hypothesis testing** | |
| --- | --- | --- | --- | --- | --- | --- |
|  | **Normal priors** | | **Regularizing priors** | |  |  |
|  | **Median (2.5%; 97.5%)** | **OR > 1** | **Median (2.5%; 97.5%)** | **OR > 1** | **Estimate (95% CI)** | **p-value** |
| **Mild vs No symptoms** | | | | | | |
| **Age** | 0.99 (0.97; 1.00) | 21.6% | 1.00 (0.97; 1.00) | 24.1% | 1.00 (0.98; 1.03) | 0.702 |
| **Man vs Woman** | 0.81 (0.62; 0.89) | 5.6% | 1.00 (0.79; 1.00) | 36.2% | 0.82 (0.63; 1.07) | 0.144 |
| **Other vs Woman** | 0.58 (0.17; 0.84) | 15.9% | 1.00 (0.60; 1.01) | 45.1% | 0.46 (0.11; 2.02) | 0.304 |
| **Handwashing with soap/alcohol*** | 1.24 (0.73; 1.48) | 79.7% | 1.00 (0.92; 1.02) | 56.9% | 1.25 (0.74; 2.11) | 0.400 |
| **Remained at home*** | 1.30 (0.97; 1.44) | 95.9% | 1.01 (0.96; 1.07) | 68.6% | 1.33 (0.99; 1.78) | 0.054 |
| **Sneezed/coughed in your arm*** | 1.35 (0.86; 1.58) | 90.3% | 1.00 (0.92; 1.02) | 57.6% | 1.40 (0.88; 2.23) | 0.151 |
| **Kept a distance from others when you have gone out*** | 1.27 (0.91; 1.43) | 92.2% | 1.00 (0.95; 1.03) | 61.6% | 1.31 (0.94; 1.84) | 0.116 |
| **Avoided meeting with persons who are older/in a risk group*** | 0.50 (0.23; 0.63) | 1.6% | 1.00 (0.45; 1.00) | 35.3% | 0.47 (0.22; 0.99) | 0.046 |
| **Avoided traveling with public transportation*** | 1.20 (0.93; 1.31) | 92.3% | 1.00 (0.95; 1.03) | 61.6% | 1.24 (0.96; 1.61) | 0.100 |
| **Avoided travel to other places in the country*** | 0.78 (0.54; 0.88) | 7.9% | 1.00 (0.81; 1.00) | 42.0% | 0.79 (0.55; 1.14) | 0.204 |
| **Moderate vs No symptoms** | | | | | | |
| **Age** | 1.05 (1.02; 1.05) | >99.9% | 1.04 (1.02; 1.05) | 99.9% | 1.07 (1.04; 1.09) | < 0.001 |
| **Man vs Woman** | 1.02 (0.78; 1.12) | 56% | 1.00 (0.89; 1.02) | 52.4% | 1.04 (0.79; 1.38) | 0.765 |
| **Other vs Woman** | 0.45 (0.12; 0.68) | 9.1% | 0.99 (0.25; 1.01) | 38.7% | 0.26 (0.03; 1.99) | 0.196 |
| **Handwashing with soap/alcohol*** | 0.90 (0.48; 1.10) | 36.4% | 1.00 (0.79; 1.02) | 47.7% | 0.89 (0.48; 1.66) | 0.720 |
| **Remained at home*** | 1.80 (1.35; 1.99) | >99.9% | 1.60 (1.05; 1.78) | 99.2% | 1.87 (1.39; 2.51) | < 0.001 |
| **Sneezed/coughed in your arm*** | 1.04 (0.61; 1.24) | 56.4% | 1.00 (0.81; 1.02) | 49.5% | 1.06 (0.63; 1.8) | 0.820 |
| **Kept a distance from others when you have gone out*** | 0.95 (0.64; 1.08) | 39.1% | 1.00 (0.8; 1.01) | 43.3% | 0.98 (0.66; 1.45) | 0.925 |
| **Avoided meeting with persons who are older/in a risk group*** | 0.80 (0.42; 0.99) | 23.8% | 1.00 (0.75; 1.01) | 45.5% | 0.80 (0.42; 1.51) | 0.494 |
| **Avoided traveling with public transportation*** | 1.19 (0.90; 1.3) | 89.2% | 1.00 (0.92; 1.04) | 59.1% | 1.25 (0.94; 1.65) | 0.121 |
| **Avoided travel to other places in the country*** | 0.86 (0.58; 0.97) | 20.2% | 1.00 (0.81; 1.01) | 44.8% | 0.88 (0.6; 1.29) | 0.525 |
| **Severe vs No symptoms** | | | | | | |
| **Age** | 1.04 (1.01; 1.05) | 99.4% | 1.02 (1.00; 1.03) | 93.7% | 1.07 (1.04; 1.1) | < 0.001 |
| **Man vs Woman** | 0.82 (0.55; 0.93) | 14.6% | 1.00 (0.73; 1.00) | 37.6% | 0.85 (0.58; 1.25) | 0.412 |
| **Other vs Woman** | 0.96 (0.26; 1.42) | 47.1% | 1.00 (0.73; 1.02) | 49.7% | 0.96 (0.22; 4.23) | 0.953 |
| **Handwashing with soap/alcohol*** | 0.37 (0.12; 0.53) | 1.8% | 0.99 (0.20; 1.00) | 32.9% | 0.27 (0.07; 1.11) | 0.069 |
| **Remained at home*** | 1.49 (1.00; 1.70) | 97.6% | 1.02 (0.95; 1.19) | 73.1% | 1.57 (1.06; 2.34) | 0.025 |
| **Sneezed/coughed in your arm*** | 1.07 (0.53; 1.33) | 57.9% | 1.00 (0.81; 1.02) | 50.5% | 1.10 (0.55; 2.2) | 0.778 |
| **Kept a distance from others when you have gone out*** | 0.76 (0.44; 0.91) | 15.2% | 1.00 (0.68; 1.01) | 40.4% | 0.79 (0.45; 1.38) | 0.410 |
| **Avoided meeting with persons who are older/in a risk group*** | 1.14 (0.54; 1.44) | 64.1% | 1.00 (0.86; 1.03) | 54.9% | 1.17 (0.56; 2.42) | 0.681 |
| **Avoided traveling with public transportation*** | 1.27 (0.89; 1.44) | 90.7% | 1.01 (0.93; 1.06) | 64.6% | 1.38 (0.96; 1.98) | 0.085 |
| **Avoided travel to other places in the country*** | 1.35 (0.86; 1.57) | 91.1% | 1.01 (0.94; 1.13) | 69.3% | 1.46 (0.94; 2.28) | 0.094 |
| **Died vs No Symptoms** | | | | | | |
| **Age** | 1.05 (1.02; 1.07) | 99.8% | 1.05 (1; 1.06) | 98.2% | 1.13 (1.08; 1.18) | < 0.001 |
| **Man vs Woman** | 0.71 (0.40; 0.86) | 10.9% | 1.00 (0.63; 1.01) | 38.0% | 0.78 (0.43; 1.42) | 0.416 |
| **Other vs Woman** | 0.97 (0.20; 1.56) | 48.1% | 1.00 (0.73; 1.02) | 49.9% | 1.04 (0.13; 8.1) | 0.968 |
| **Handwashing with soap/alcohol*** | 0.76 (0.22; 1.10) | 30.6% | 1.00 (0.66; 1.01) | 46.2% | 0.74 (0.17; 3.13) | 0.678 |
| **Remained at home*** | 1.24 (0.65; 1.52) | 74.8% | 1.00 (0.83; 1.02) | 51.4% | 1.39 (0.73; 2.65) | 0.314 |
| **Sneezed/coughed in your arm*** | 1.15 (0.44; 1.54) | 62.4% | 1.00 (0.80; 1.02) | 51.5% | 1.19 (0.45; 3.13) | 0.722 |
| **Kept a distance from others when you have gone out*** | 0.68 (0.27; 0.90) | 17.5% | 1.00 (0.56; 1.01) | 40.6% | 0.74 (0.29; 1.91) | 0.536 |
| **Avoided meeting with persons who are older/in a risk group*** | 1.23 (0.43; 1.69) | 65.8% | 1.00 (0.82; 1.03) | 52.6% | 1.37 (0.48; 3.91) | 0.562 |
| **Avoided traveling with public transportation*** | 0.62 (0.33; 0.76) | 5.3% | 0.98 (0.44; 1.00) | 28.3% | 0.72 (0.38; 1.37) | 0.319 |
| **Avoided travel to other places in the country*** | 0.73 (0.31; 0.96) | 21.8% | 1.00 (0.64; 1.01) | 43.2% | 0.84 (0.35; 2.01) | 0.688 |
| **Not relevant/Do not know vs No symptoms** | | | | | | |
| **Age** | 1.04 (1.03; 1.05) | >99.9% | 1.04 (1.02; 1.04) | >99.9% | 1.05 (1.04; 1.07) | < 0.001 |
| **Man vs Woman** | 1.18 (0.99; 1.26) | 96.4% | 1.09 (0.98; 1.18) | 87.4% | 1.20 (1.00; 1.44) | 0.051 |
| **Other vs Woman** | 1.64 (0.85; 2.05) | 93.3% | 1.01 (0.91; 1.11) | 66.3% | 1.59 (0.80; 3.15) | 0.187 |
| **Handwashing with soap/alcohol*** | 0.91 (0.61; 1.04) | 32.3% | 1.00 (0.83; 1.01) | 47.3% | 0.90 (0.60; 1.35) | 0.598 |
| **Remained at home*** | 0.94 (0.75; 1.01) | 28.4% | 0.99 (0.81; 1.00) | 34.5% | 0.96 (0.76; 1.19) | 0.689 |
| **Sneezed/coughed in your arm*** | 1.04 (0.74; 1.17) | 58.7% | 1.00 (0.87; 1.02) | 49.7% | 1.05 (0.74; 1.48) | 0.794 |
| **Kept a distance from others when you have gone out*** | 1.25 (0.98; 1.36) | 96.7% | 1.04 (0.96; 1.15) | 78.5% | 1.28 (1; 1.63) | 0.053 |
| **Avoided meeting with persons who are older/in a risk group*** | 0.94 (0.64; 1.08) | 38.6% | 1.00 (0.86; 1.02) | 51.3% | 0.93 (0.62; 1.39) | 0.716 |
| **Avoided traveling with public transportation*** | 1.29 (1.07; 1.38) | 99.7% | 1.06 (0.98; 1.14) | 83.3% | 1.32 (1.10; 1.60) | 0.003 |
| **Avoided travel to other places in the country*** | 0.85 (0.66; 0.93) | 11.2% | 1.00 (0.84; 1.01) | 40.7% | 0.87 (0.68; 1.12) | 0.279 |
| * Non-compliant vs Compliant | | | | | | |
