# Supplementary material for: Associations between compliance with covid-19 public health recommendations and perceived contagion in others: a self-report study in Swedish university students
Source: BMC Res Notes. 2021 Nov 25;14:429. doi: 10.1186/s13104-021-05848-6 (PMC8613723; doi:10.1186/s13104-021-05848-6)
Supplement: Supplementary file 9 — Additional file 9: Table S9. Symptoms of contagion in another person and self-reported recommendation compliance—contingency table. [file 13104_2021_5848_MOESM9_ESM.docx]

Table S9. Symptoms of contagion in another person and self-reported recommendation compliance – Contingency table.

| **Self-reported symptoms of another person vs recommendation compliance** | | | | | | |
| --- | --- | --- | --- | --- | --- | --- |
|  | **No symptoms** | **Mild symptoms** | **Moderate symptoms** | **Severe symptoms** | **Died** | **Not relevant/Do not know** |
| **Handwashing with soap/alcohol** | | | | | | |
| **Compliance** | 982 (95.2%) | 54 (94.7%) | 107 (98.2%) | 83 (96.5%) | 146 (98.6%) | 1102 (95.3%) |
| **Non-compliance** | 50 (4.8%) | 3 (5.3%) | 2 (1.8%) | 3 (3.5%) | 2 (1.4%) | 54 (4.7%) |
| **Remained at home** | | | | | | |
| **Compliance** | 846 (81.9%) | 42 (73.7%) | 83 (76.1%) | 72 (83.7%) | 127 (85.8%) | 952 (82.4%) |
| **Non-compliance** | 187 (18.1%) | 15 (26.3%) | 26 (23.9%) | 14 (16.3%) | 21 (14.2%) | 204 (17.6%) |
| **Sneezed/coughed in your arm** | | | | | | |
| **Compliance** | 968 (94%) | 51 (89.5%) | 100 (91.7%) | 82 (95.3%) | 135 (91.8%) | 1080 (93.6%) |
| **Non-compliance** | 62 (6%) | 6 (10.5%) | 9 (8.3%) | 4 (4.7%) | 12 (8.2%) | 74 (6.4%) |
| **Kept a distance from others when you have gone out** | | | | | | |
| **Compliance** | 901 (87.2%) | 47 (82.5%) | 100 (91.7%) | 78 (90.7%) | 138 (93.2%) | 1007 (87.1%) |
| **Non-compliance** | 132 (12.8%) | 10 (17.5%) | 9 (8.3%) | 8 (9.3%) | 10 (6.8%) | 149 (12.9%) |
| **Avoided meeting with persons who are older/in a risk group** | | | | | | |
| **Compliance** | 991 (95.9%) | 53 (93%) | 103 (95.4%) | 82 (95.3%) | 144 (97.3%) | 1104 (95.5%) |
| **Non-compliance** | 42 (4.1%) | 4 (7%) | 5 (4.6%) | 4 (4.7%) | 4 (2.7%) | 52 (4.5%) |
| **Avoided traveling with public transportation** | | | | | | |
| **Compliance** | 700 (67.8%) | 41 (71.9%) | 80 (73.4%) | 62 (72.1%) | 114 (77%) | 794 (68.7%) |
| **Non-compliance** | 332 (32.2%) | 16 (28.1%) | 29 (26.6%) | 24 (27.9%) | 34 (23%) | 362 (31.3%) |
| **Avoided travel to other places in the country** | | | | | | |
| **Compliance** | 888 (86.1%) | 51 (89.5%) | 92 (85.2%) | 79 (91.9%) | 132 (89.2%) | 1021 (88.5%) |
| **Non-compliance** | 143 (13.9%) | 6 (10.5%) | 16 (14.8%) | 7 (8.1%) | 16 (10.8%) | 133 (11.5%) |
