# Supplementary figures and images for: Associations between compliance with covid-19 public health recommendations and perceived contagion in others: a self-report study in Swedish university students
Source: BMC Res Notes. 2021 Nov 25;14:429. doi: 10.1186/s13104-021-05848-6 (PMC8613723; doi:10.1186/s13104-021-05848-6)

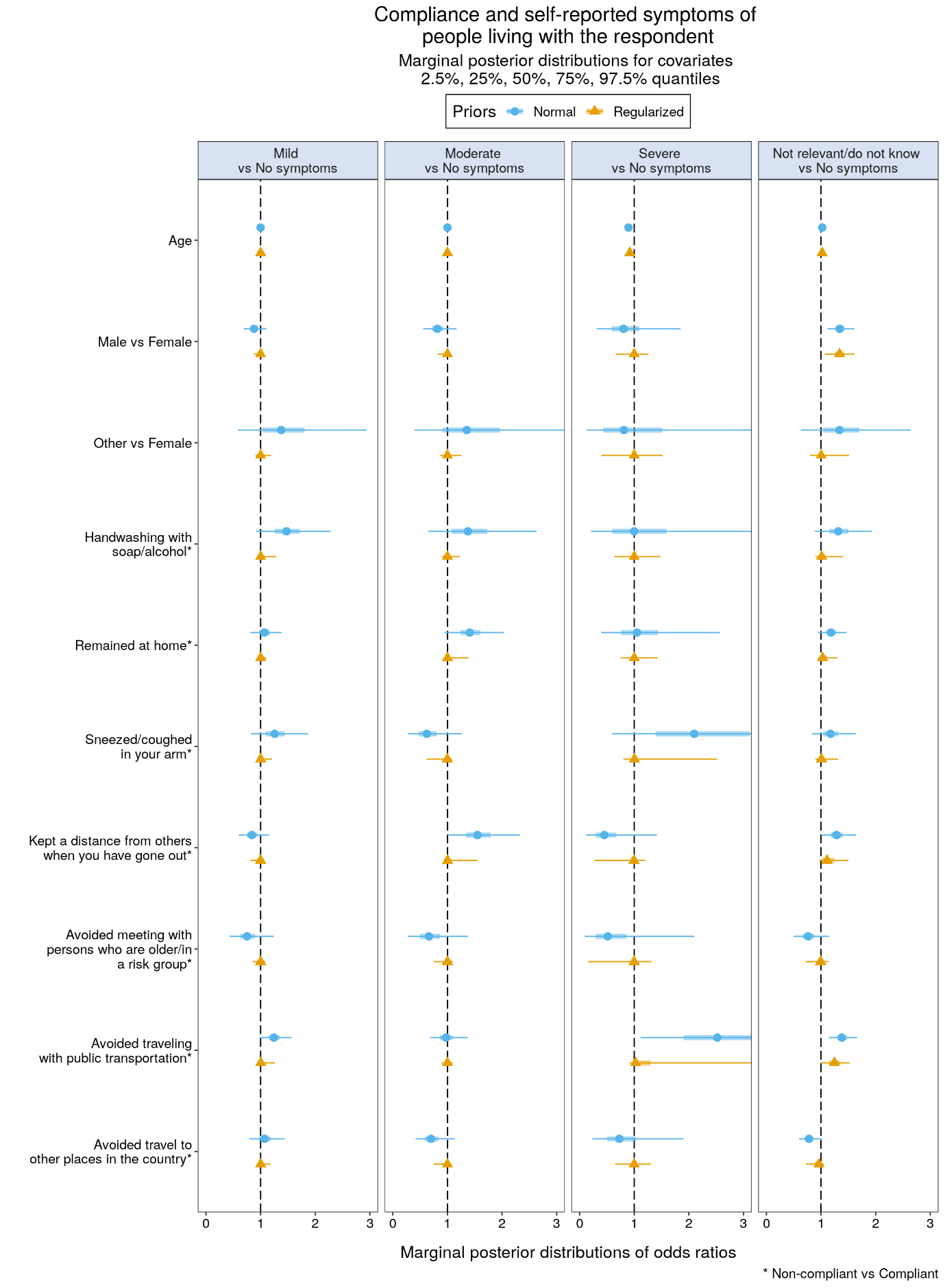


Figure S1. Compliance and self-reported symptoms of people living with the respondent.

Supplement: Supplementary file 11 — Additional file 11: Figure S1. Compliance and self-reported symptoms of people living with the respondent. [file 13104_2021_5848_MOESM11_ESM.docx]

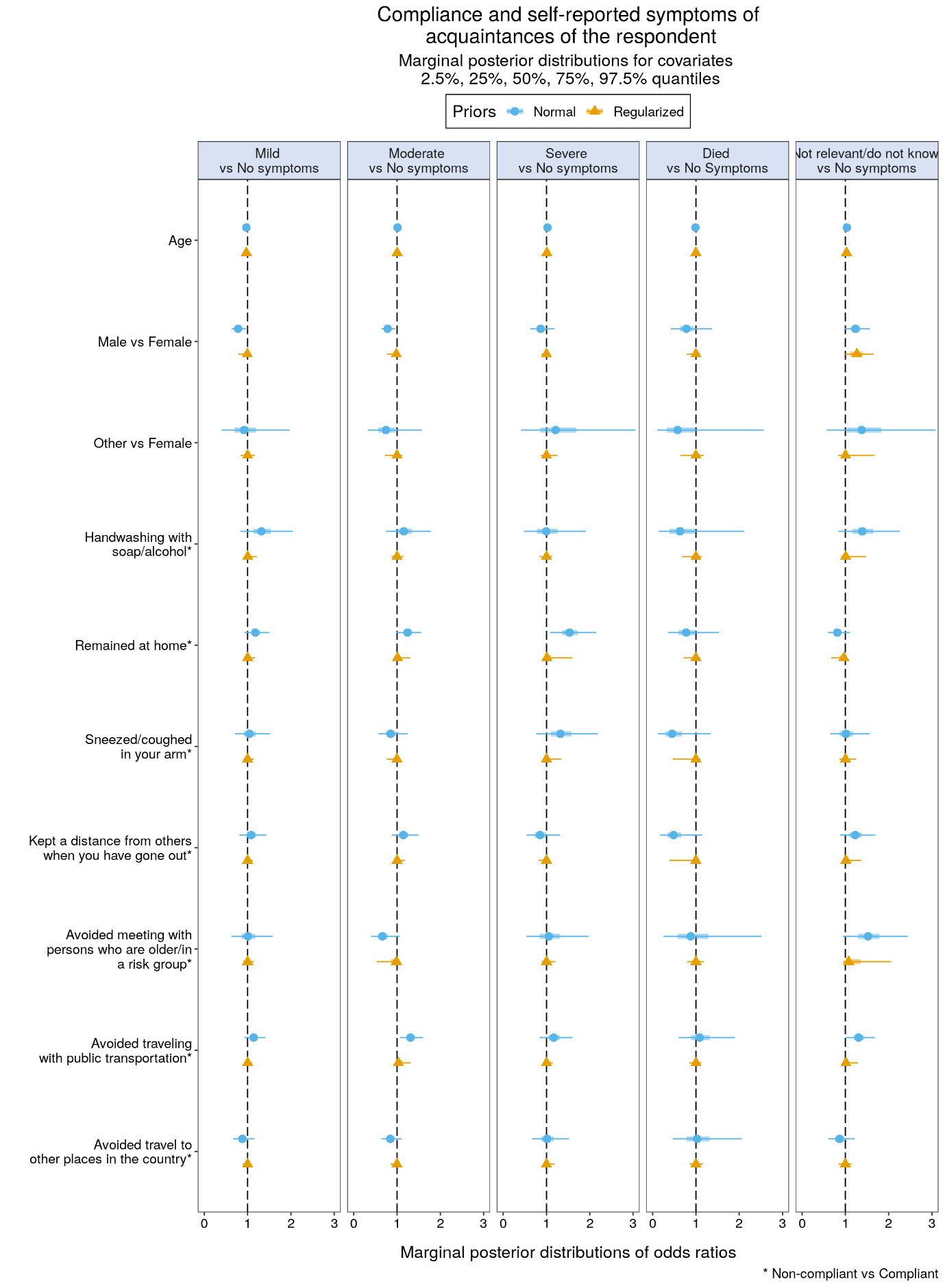


Figure S3. Compliance and self-reported symptoms of acquaintances of the respondent.

Supplement: Supplementary file 13 — Additional file 13: Figure S3. Compliance and self-reported symptoms of acquaintances of the respondent. [file 13104_2021_5848_MOESM13_ESM.docx]

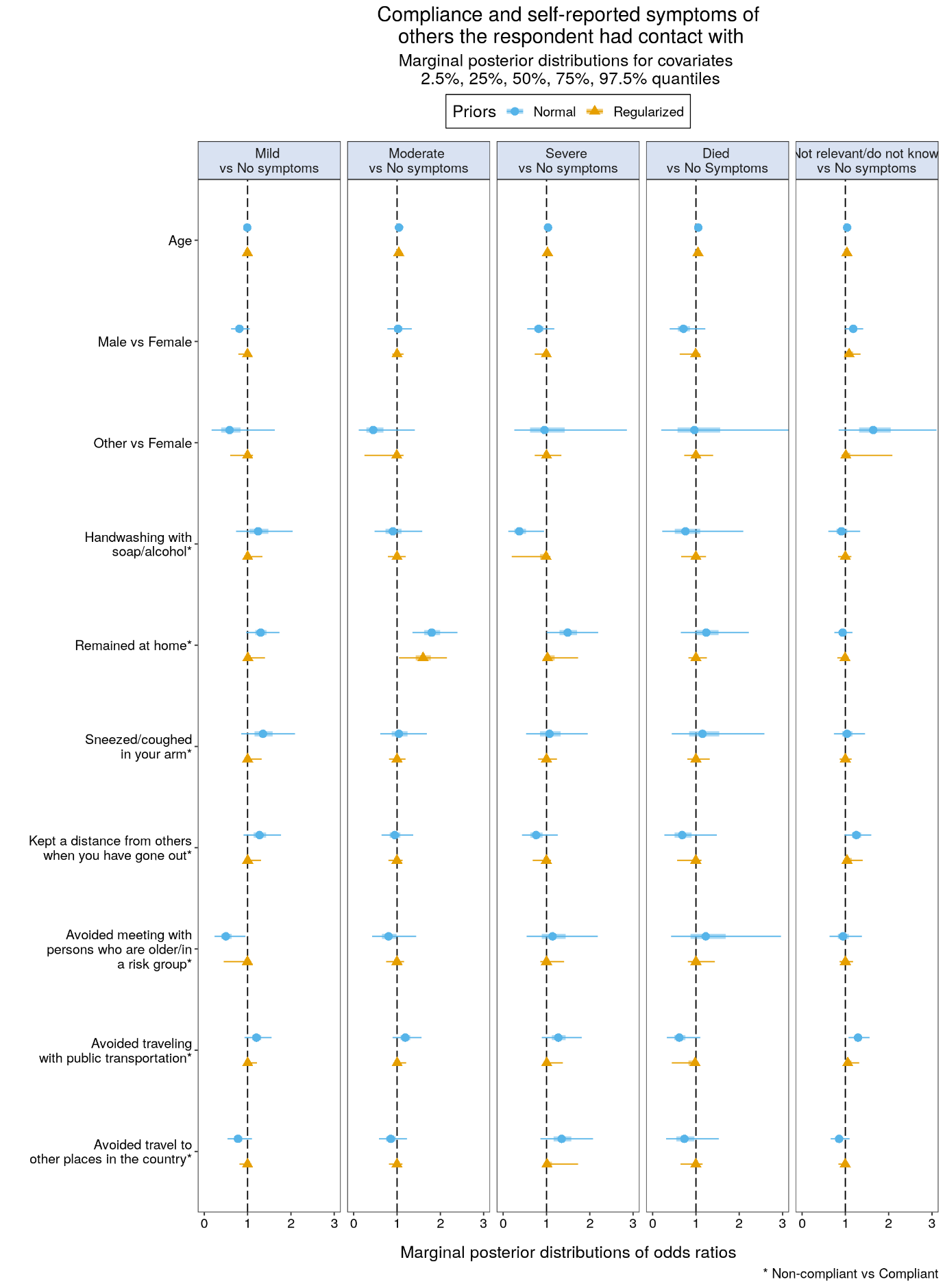


Figure S4. Compliance and self-reported symptoms of others the respondent had contact with.

Supplement: Supplementary file 14 — Additional file 14: Figure S4. Compliance and self-reported symptoms of others the respondent had contact with. [file 13104_2021_5848_MOESM14_ESM.docx]

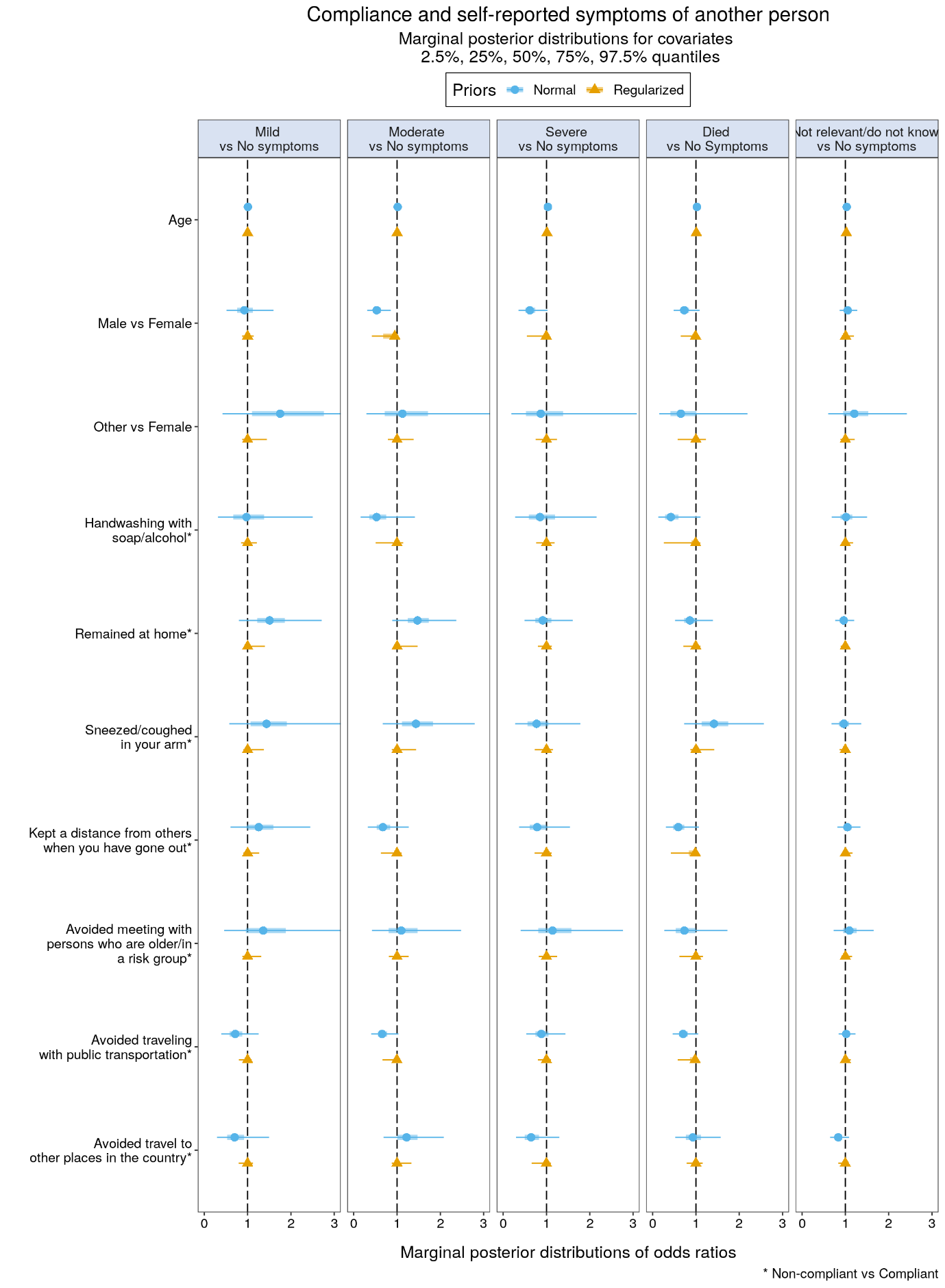


Figure S5. Compliance and self-reported symptoms of another person.

Supplement: Supplementary file 15 — Additional file 15: Figure S5. Compliance and self-reported symptoms of another person. [file 13104_2021_5848_MOESM15_ESM.docx]
