# Supplementary material for: Associations between compliance with covid-19 public health recommendations and perceived contagion in others: a self-report study in Swedish university students
Source: BMC Res Notes. 2021 Nov 25;14:429. doi: 10.1186/s13104-021-05848-6 (PMC8613723; doi:10.1186/s13104-021-05848-6)
Supplement: Supplementary file 12 — Additional file 12: Figure S2. Compliance and self-reported symptoms of family members of the respondent (not living with). [file 13104_2021_5848_MOESM12_ESM.docx]

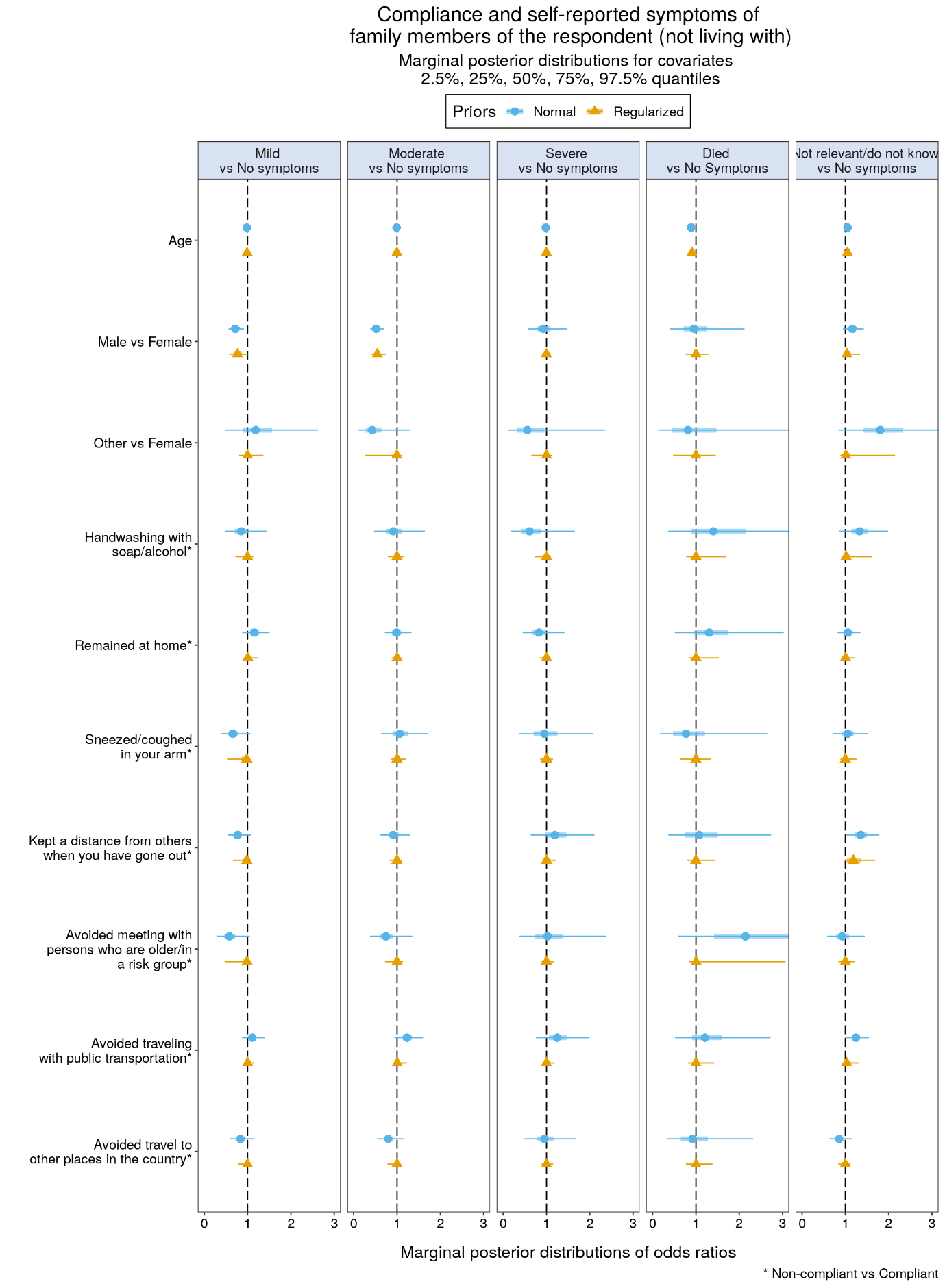


Figure S2. Compliance and self-reported symptoms of family members of the respondent (not living with).
