# Supplementary material for: Associations between compliance with covid-19 public health recommendations and perceived contagion in others: a self-report study in Swedish university students
Source: BMC Res Notes. 2021 Nov 25;14:429. doi: 10.1186/s13104-021-05848-6 (PMC8613723; doi:10.1186/s13104-021-05848-6)
Supplement: Supplementary file 16 — Additional file 16. COVID-19 questions-general link national or international. [file 13104_2021_5848_MOESM16_ESM.docx]

COVID-19 questions - general link national or international

Start of Block: COVID-19 frågor svenska

Q23
 Hi!

 Many thanks for your interest in answering questions about how the COVID-19 pandemic affects you. Your participation in this survey will contribute to generating knowledge about university students' situation and what kind of support they might need. 
 You will find more information about the study and an option to consent to participate on the next page. The survey will follow and takes about 5-10 minutes to answer.

 Best wishes,
 Anne H Berman, PhD, Principal Investigator, Lic. psychologist, lic. psychotherapist 
 Senior lecturer, Dept of Psychology,  Uppsala University, anne.h.berman@psyk.uu.se
 Claes Andersson, PhD, Associate Professor, Malmö University, claes.andersson@mau.se
 Karin Engström, PhD, Associate Professor, Swedish Public Health Agency, karin.engstrom@folkhalsomyndigheten.se
 Petra Lindfors, PhD, Professor, Stockholm University, pls@psychology.su.se
 Marcus Bendtsen, PhD, Senior Lecturer, Linköping University, marcus.bendtsen@liu.se
 Philip Lindner, PhD, Lic. psychologist, researcher, Karolinska Institutet, philip.lindner@ki.se
Naira Topooco, PhD, post-doctoral researcher, Linköping University, naira.topooco@liu.se
 Lilian Granlund, Clinical Psychology candidate, Uppsala University
 Diana Abou-Soultan, Clinical Psychology candidate, Uppsala University

 E-mail for questions about the study: swedishunisurveys@uu.se 

| Page Break |  |
| --- | --- |

Q24 **COVID-19 effects among university students** Hi! You have shown interest in participating in a study on the COVID-19 pandemic’s effects among university students. Below you will find information on the study and what your participation involves. On the next page you will find the survey questions. **What this research project is about and why we want you to participate** The purpose of this study is to investigate how the COVID-19 pandemic affects the health and studies of university students, right now and after 3 and 6 months. The follow-up is part of the study in order to contribute to knowledge about how the effects change over time. At participating universities, this study is part of a collaboration with the university leadership, the student mental health services and the student unions. The responsible research institution for this study is Uppsala University.   **What does my participation involve?** You answer a survey that takes about 5-10 minutes to respond to. If you are willing to provide your e-mail address, you will receive an invitation to a follow-up survey with the same questions, after 3 and 6 months. **Possible consequences and risks of participating in the study** The survey contains questions on your mental health, thus constituting a violation of your privacy. If the content of the survey raises concerns regarding your mental health, we recommend that you seek counseling and support via national chat and telephone helplines, by contacting the Student mental health center at your institution, or the healthcare system directly. Or, you could contact the persons responsible for this study. **What will happen to the information I provide?** Your responses and results are managed such that no outside persons will access them. Your personal data are handled according to the legal principle of “General interest” and are shielded by confidentiality. The party responsible for your data is Uppsala University. Your anonymous responses are retained in electronic systems according to the Uppsala University guidelines for research documentation, which fulfill the requirements stipulated in the EU data protection regulation. According to this regulation (GDPR) you have the right to access study data concerning your person without charge, and you have the right to rectify any incorrect data if needed. You also have the right to request that information about you be deleted, and that management of your data be limited. If you want to know more, please contact the Principal Investigator (see contact information below). You can reach the Data Protection Ombudsman at dataskyddsombud@uu.se. If you are dissatisfied with how information about you is being managed, you have the right to enter a complaint with the Data Inspection Authority, which is the authority with oversight in this case. All those working in this study are covered by a rule of confidentiality. **How do I obtain information on the study results?** When the study results are analyzed, they will be presented on a group level, meaning that no one will be able to identify what you, in particular, have answered. Study results will be published in scientific articles, reports and at scientific congresses. The results will contribute to knowledge about university students’ situation and what type of support they might need in connection with the pandemic and other similar events which might occur. **Insurance and compensation** Not relevant **Voluntary participation** Your participation is voluntary. If you choose not to participate you do not need to explain why. **Researchers responsible for the study** If you want to know more about the study, please contact the researchers below, Anne H Berman, PhD, Principal Investigator, lic. psychologist, lic. psychotherapist
 Senior Lecturer, Dept. of Psychology, Uppsala University, anne.h.berman@psyk.uu.se 018-471 6260  Claes Andersson, PhD, Malmö University, Health and Society, claes.andersson@mau.se 070-275 12 46
 Petra Lindfors, PhD, Professor of Psychology, Stockholm University, pls@psychology.su.se
 Karin Engström, PhD, Associate Professor, Evaluator, Swedish Public Health Agency, karin.engstrom@folkhalsomyndigheten.se
 Marcus Bendtsen, PhD, University Lecturer, Linköping University, marcus.bendtsen@liu.se Philip Lindner, PhD, licensed psychologist, Karolinska Institutet, philip.lindner@ki.se

| Page Break |  |
| --- | --- |

Q49 **Please indicate your willingness to participate by choosing one of the options below.**

- **I consent** to answer these questions now and am **willing to answer again after 3 and 6 months** (please provide your e-mail address) (1)
- **I consent** to answer these question now but **do not want to be contacted for follow-ups** (2)
- I do not want to answer the questions and am not providing my consent (3)

Skip To: End of Survey If Please indicate your willingness to participate by choosing one of the options below.  = I do not want to answer the questions and am not providing my consent

| Page Break |  |
| --- | --- |

Display This Question:

If Please indicate your willingness to participate by choosing one of the options below.  = <u><strong>I consent</strong></u> to answer these questions now and am <u><strong>willing to answer again after 3 and 6 months</strong></u> (please provide your e-mail address)

| 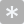 |
| --- |

Q25 I am hereby providing my e-mail address for follow-up after 3 and 6 months

________________________________________________________________

| Page Break |  |
| --- | --- |

| 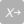 | 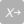 |
| --- | --- |

Q26 **What is your current gender identity?**

- Man (1)
- Woman (2)
- Other (please describe briefly in the text box) (3) ________________________________________________

| 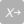 | 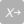 |
| --- | --- |

Q27 **How old are you?**

- 16 or younger (16)
- 17 (17)
- 18 (18)
- 19 (19)
- 20 (20)
- 21 (21)
- 22 (22)
- 23 (23)
- 24 (24)
- 25 (25)
- 26 (26)
- 27 (27)
- 28 (28)
- 29 (29)
- 30 (30)
- 31 (31)
- 32 (32)
- 33 (33)
- 34 (34)
- 35 (35)
- 36 or older (36)

| Page Break |  |
| --- | --- |

Q28 Which university/college are you studying at?

- Gothenburg University (1)
- Högskolan i Gävle (15)
- Högskolan i Halmstad (13)
- Royal College of Music (5)
- Linné University (2)
- Luleå tekniska universitet (10)
- Mittuniversitetet (11)
- Malmö universitet (12)
- Umeå University (7)
- Uppsala University (8)
- Other university/college, please indicate (9) ________________________________________________

| Page Break |  |
| --- | --- |

Q29 What is the primary academic area you are studying?

- Humanities and theology (1)
- Medicine and dentistry (2)
- Law and social sciences (3)
- Healthcare professions (4)
- Natural sciences (5)
- Fine arts (6)
- Engineering and technology (7)
- Other (9)

End of Block: COVID-19 frågor svenska

Start of Block: COVID-19 questions English

| Page Break |  |
| --- | --- |

| 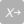 | 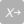 |
| --- | --- |

Q3 **The Public Health Agency has recommended several behaviors for reducing the spread of COVID-19. To what extent have you kept to the recommendations during the past 4 weeks?**

|  | Often, very often, or always (1) | Less often, seldom or never (2) |
| --- | --- | --- |
| Handwashing with soap/alcohol (COVID19beh_1) |  |  |
| Remained at home (COVID19beh_2) |  |  |
| Sneezed/coughed in your arm (COVID19beh_3) |  |  |
| Kept a distance from others when you have gone out (COVID19beh_4) |  |  |
| Avoided meeting with persons who or older/in a risk group (COVID19beh_5) |  |  |
| Avoided traveling with public transportation (COVID19beh_6) |  |  |
| Avoided travel to other places in Sweden (COVID19troth) |  |  |

| Page Break |  |
| --- | --- |

Display This Question:

If The Public Health Agency has recommended several behaviors for reducing the spread of COVID-19. T... [ Less often, seldom or never] (Count) >= 1

Q4 **You have indicated that you have followed one or more of the recommendations to a lesser extent. Why do you think that is?**

|  | Yes (1) | No (2) |
| --- | --- | --- |
| Deficient communication from authorities (1) |  |  |
| Deficient scientific evidence (2) |  |  |
| Deficient trust in the method for reducing the spread of contagion (3) |  |  |
| Because my finances are worse (4) |  |  |
| Social pressure (e.g., others around me do not follow the recommendations (5) |  |  |
| I do not want to limit my freedom (6) |  |  |
| I work in the healthcare sector (7) |  |  |
| I have already had COVID-19 and have recovered (8) |  |  |

| Page Break |  |
| --- | --- |

Q5 **To what extent have you been affected in a tangible way by the COVID19 pandemic?**

- No symptoms (2)
- Mild symptoms (3)
- Moderate symptoms (4)
- Severe symptoms (5)
- Not relevant/do not know (1)

| Page Break |  |
| --- | --- |

Display This Question:

If To what extent have you been affected in a tangible way by the COVID19 pandemic? = Mild symptoms

Or To what extent have you been affected in a tangible way by the COVID19 pandemic? = Moderate symptoms

Or To what extent have you been affected in a tangible way by the COVID19 pandemic? = Severe symptoms

Q6 You indicated that you have experienced COVID19 symptoms. What sort of help have you sought and to what extent did you receive adequate help?

|  | I have not sought this type of care (1) | I sought this type of care and received adequate help (2) | I sought this type of care but did not receive adequate help (3) |
| --- | --- | --- | --- |
| Help via web/app/telephone (2) |  |  |  |
| Primary care (4) |  |  |  |
| Specialist care (7) |  |  |  |
| Hospital care (8) |  |  |  |
| Other, please indicate (9) |  |  |  |

| Page Break |  |
| --- | --- |

Q7 **To what extent have the following persons, with whom you have had contact, been tangibly affected by the COVID-19 pandemic?**

|  | No symptoms (1) | Mild symptoms (2) | Moderate symptoms (3) | Severe symptoms (4) | Died (5) | Not relevant/do not know (6) |
| --- | --- | --- | --- | --- | --- | --- |
| A person I live with (1) |  |  |  |  |  |  |
| A person in my family (not living with) (2) |  |  |  |  |  |  |
| A person in my circle of acquaintances (3) |  |  |  |  |  |  |
| Someone else I have had contact with (4) |  |  |  |  |  |  |
| Another person, describe if you wish: (6) |  |  |  |  |  |  |

| Page Break |  |
| --- | --- |

| 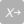 |
| --- |

Q8 **How has your mental health been affected by the COVID-19 pandemic during the past 4 weeks?**

- No effect (1)
- My mental health has been worse (2)
- My mental health has been better (3)
- My mental health has been both better and worse (4)

| Page Break |  |
| --- | --- |

Display This Question:

If How has your mental health been affected by the COVID-19 pandemic during the past 4 weeks? = My mental health has been worse

Or How has your mental health been affected by the COVID-19 pandemic during the past 4 weeks? = My mental health has been both better and worse

| 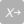 |
| --- |

Q9 **You indicated that your mental health during the past 4 weeks has been worse.**

- This worsening is entirely or partly due to the COVID-19 pandemic (1)
- The worsening is due to factors that have nothing to do with the COVID-19 pandemic (2)

| Page Break |  |
| --- | --- |

Display This Question:

If You indicated that your mental health during the past 4 weeks has been worse. = This worsening is entirely or partly due to the COVID-19 pandemic

Q10 **You indicated that your mental health during the past 4 weeks has been worse, and that this worsening is entirely or partly due to the COVID-19 pandemic. Which mental health symptoms have you had as a result of the COVID-19 pandemic? (You may indicate several responses.)**

- Worry or anxiety (1)
- Depression or low mood (2)
- Difficulty sleeping (3)
- Stress (4)
- Other, please indicate: (5) ________________________________________________

| Page Break |  |
| --- | --- |

Display This Question:

If You indicated that your mental health during the past 4 weeks has been worse. = This worsening is entirely or partly due to the COVID-19 pandemic

| 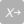 | 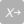 |
| --- | --- |

Q11 You indicated that your mental health during the past 4 weeks has been worse, and that this worsening is entirely or partly due to the COVID-19 pandemic. In what way/s has the COVID-19 pandemic worsened your mental health?  (You may indicate several responses.)

- I have felt lonely/isolated (1)
- It has been more difficult to study at home or remotely (2)
- I have been worried about falling ill (3)
- I belong to a risk group (4)
- I have been worried that friends, acquaintances or relatives will fall ill (5)
- Uncertainty about what will happen in society has affected me negatively (6)
- Worry and uncertainty about whether I will complete my studies/obtain my degree in time (7)
- Worry about how the COVID-19 pandemic will affect my finances. (8)
- Other, please indicate: (9) ________________________________________________

| Page Break |  |
| --- | --- |

Display This Question:

If How has your mental health been affected by the COVID-19 pandemic during the past 4 weeks? = My mental health has been better

Or How has your mental health been affected by the COVID-19 pandemic during the past 4 weeks? = My mental health has been both better and worse

Q12 You indicated that your mental health during the past 4 weeks has been better.

- This improvement is entirely or partly due to the COVID-19 pandemic (1)
- This improvement is due to factors that have nothing to do with the COVID-19 pandemic (2)

Display This Question:

If You indicated that your mental health during the past 4 weeks has been better. In what way/s has ... = This improvement is entirely or partly due to the COVID-19 pandemic

Q13 You indicated that your mental health during the past 4 weeks has been better, and that this improvement is entirely or partly due to the COVID-19 pandemic. Which mental health symptoms have you had as a result of the COVID-19 pandemic? (You may indicate several responses.)

- I have not been as worried as usual (1)
- I am happier or more satisfied than I usually am (2)
- My sleep has improved (3)
- I feel calmer, less stressed (4)
- Other, please indicate: (5) ________________________________________________

| Page Break |  |
| --- | --- |

Display This Question:

If You indicated that your mental health during the past 4 weeks has been better. In what way/s has ... = This improvement is entirely or partly due to the COVID-19 pandemic

Q14 You indicated that your mental health during the past 4 weeks has been better, and that this improvement is entirely or partly due to the COVID-19 pandemic. In what way/s has the COVID-19 pandemic improved your mental health?  (You may indicate several responses.)

- I have stayed at home and therefore feel less stressed (1)
- It is easier for me to study at home, or remotely, compared to usual classroom teaching. (2)
- When there is a crisis in society my problems seem less severe (3)
- The feeling of meaningfulness from the importance of what I am doing (e.g., staying at home to minimize contagion, or helping someone) (4)
- The feeling of community and togetherness (e.g., in society or with friends, relatives, neighbors (5)
- In comparison with others in society, I have felt privileged as a student (e.g., because I have been able to continue studying during this time, am not dependent on a salary/work, or any other reason). (6)
- Other please indicate: (7) ________________________________________________

| Page Break |  |
| --- | --- |

| 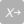 |
| --- |

Q15 **How have your studies been going during the past 4 weeks?**

- No change, my studies are going as usual (1)
- My studies have been going worse (2)
- My studies have been going better (3)
- My studies have been going both better and worse (in differing ways) (4)
- I am not studying at this time; please comment: (5) ________________________________________________

| Page Break |  |
| --- | --- |

Display This Question:

If How have your studies been going during the past 4 weeks? = My studies have been going worse

Or How have your studies been going during the past 4 weeks? = My studies have been going both better and worse (in differing ways)

Q16 **You indicated that your studies have been negatively affected by the COVID-19 pandemic. In what way/s has the pandemic affected your studies negatively? (You can indicate several answers.)**

- It has been harder for me to concentrate on my studies (1)
- Studying at home is hard for me (2)
- Online teaching has not worked well for me (3)
- My university/college has not adapted teaching during this time for it to work well enough (4)
- I have lost out on important components of teaching (e.g., due to cancellation). (5)
- Less contact with my fellow students has affected my studies negatively (6)
- Worry about not completing my education in time has affected my studies negatively (7)
- Worry about my own or others' health has affected my studies negatively (8)
- Other, please indicate: (9) ________________________________________________

| Page Break |  |
| --- | --- |

Display This Question:

If How have your studies been going during the past 4 weeks? = My studies have been going better

Or How have your studies been going during the past 4 weeks? = My studies have been going both better and worse (in differing ways)

Q17 You indicated that your studies have been positively affected by the COVID-19 pandemic. In what way/s has the pandemic affected your studies positively? (You can choose several answers.)

- It has been easier for me to concentrate on my studies (1)
- Studying at home is easier for me than studying on campus/at the university/college (2)
- Teaching via the internet has worked well for me (e.g., online lectures) (3)
- The teaching adaptations at my university/college during this time have worked well for me (4)
- I have avoided tough aspects related to teaching (e.g., due to cancellation) (5)
- Less contact with my fellow students has affected my studies positively (6)
- I feel less pressure to finish my education on time (7)
- Worry over my own or others' health has led to less worry over my studies (8)
- Other, please indicate: (9) ________________________________________________

| Page Break |  |
| --- | --- |

Q18 **How have you experienced your university's/college's way of managing the COVID-19 pandemic? (Please choose one response.)**

- Excellent (1)
- Good (2)
- Moderate (3)
- Bad (4)
- Very bad (5)
- Please motivate your response: (6) ________________________________________________

| Page Break |  |
| --- | --- |

Q19 **Based on your experience of the COVID-19 pandemic during this spring, do you have any ideas or suggestions about what your university/college, or your student mental health services, could do to help or facilitate matters for students during times of crisis, or in general?**

- I have no suggestions (1)
- The university/college should provide clearer information in such situations (2)
- The university/college should be prepared for such situations (e.g., in terms of adapted premises, providing protective gear for students in clinical practicums) (3)
- Better solutions for online/remote teaching need to be available in case of crises of different kinds (4)
- Internet-based teaching should be part of ordinary teaching (e.g., online lectures) (5)
- The student mental health services should be better prepared to offer mental health support in such situations (6)
- Other, please indicate: (7) ________________________________________________

| Page Break |  |
| --- | --- |

Q20 **The situation in connection with the COVID-19 pandemic can be handled in different ways. Please indicate how often the following thoughts and feelings about the pandemic occur for you.**

|  | (almost) never (1) | sometimes (2) | usually (3) | often (4) | (almost) always (5) |
| --- | --- | --- | --- | --- | --- |
| I often think about how I feel about what I have been experiencing (CERQ8_2) |  |  |  |  |  |
| I am preoccupied with what I think and feel about what I have been experiencing (CERQ8_6) |  |  |  |  |  |
| I think of pleasant things that have nothing to do with what is happening (CERQ8_7) |  |  |  |  |  |
| I keep thinking about how terrible what I have experienced is (CERQ8_9) |  |  |  |  |  |
| I think of something nice instead of what has happened (CERQ8_11) |  |  |  |  |  |
| I think that this hasn’t been too bad compared to other things (CERQ8_13) |  |  |  |  |  |
| I tell myself that there are worse things in life (CERQ8_16) |  |  |  |  |  |
| I continually think how horrible the situation has been (CERQ8_17) |  |  |  |  |  |

| Page Break |  |
| --- | --- |

Q21 Please share your comments on thoughts, reflections or experiences that have come up for you while answering these questions.

________________________________________________________________

________________________________________________________________

________________________________________________________________

________________________________________________________________

________________________________________________________________

End of Block: COVID-19 questions English
